# Phenolic profile of a Parma violet unveiled by chemical and fluorescence imaging

**DOI:** 10.1093/aobpla/plab041

**Published:** 2021-07-06

**Authors:** Moustafa Khatib, Cécile Pouzet, Claude Lafitte, Justine Chervin, Valérie Bonzon-Ponnet, Alain Jauneau, Marie-Thérèse Esquerré-Tugayé

**Affiliations:** 1 Laboratoire de Recherche en Sciences Végétales, Université de Toulouse, CNRS, UPS, 31326 Castanet-Tolosan, France; 2 Plateforme Imagerie FRAIB-TRI, Université de Toulouse, CNRS, 31326 Castanet-Tolosan, France; 3 Plateforme MetaToul-AgromiX, Université de Toulouse, CNRS, 31326 Castanet-Tolosan, France; 4 Groupe Berdoues, 131 Route de Toulouse, BP 10 006, 31270 Cugnaux, France

**Keywords:** Autofluorescence, chemical quantification, confocal microscopy, Parma violet, phenolic profile, *Viola alba* subsp. *dehnhardtii*

## Abstract

The ability of phenolic compounds to autofluoresce upon illumination by UV or blue light was exploited to explore the nature and distribution of these metabolites within the flower petals, leaves and roots of the violet, *Viola alba* subsp. *dehnhardtii*. This was achieved through a dual complementary approach that combined fluorescence microscopy imaging of living intact tissues and chemical extraction of pulverized material. The blue to red fluorescence displayed by living tissues upon illumination was indicative of their richness in phenolic compounds. Phenolic acids were found in all tissues, while flavonoids characterized the aerial part of the plant, anthocyanidins being restricted to the petals. The chemical quantification of phenolics in plant extracts confirmed their tissue-specific distribution and abundance. A key finding was that the spectral signatures obtained through confocal microscopy of endogenous fluorophores in living tissues and their counterpart extracts share the same fluorescence patterns, pointing out the potential of fluorescence imaging of intact organs for a proper estimation of their phenolic content. In addition, this study highlighted a few distinct morphology cell types, in particular foliar-glandular-like structures, and jagged petal cell walls. Altogether, these data provide a comprehensive histochemical localization of phenolics in living tissues of a violet. Converting fluorescence imaging into a chemical imprint indicated that one can rely on fluorescence microscopy of intact living tissues as a rapid, non-destructive means to follow their phenolic imprint under various environmental conditions.

## Teaser

The Parma violet *Viola alba* subsp. *dehnhardtii* distinguishes from other violets by the presence of double chasmogame flowers harbouring up to 40 petals. The phenolic imprint of its different plant parts was characterized by a dual approach that combines fluorescence imaging of living tissues and chemical analysis. The knowledge gained from this study indicated that one can rely on fluorescence microscopy as a rapid, non-destructive means to follow the phenolic pattern of a given organ or tissue. It also highlighted a few distinct morphology cell types, most notably foliar-glandular-like structures, and jagged petal cell walls.

## Introduction

**Figure 1.**
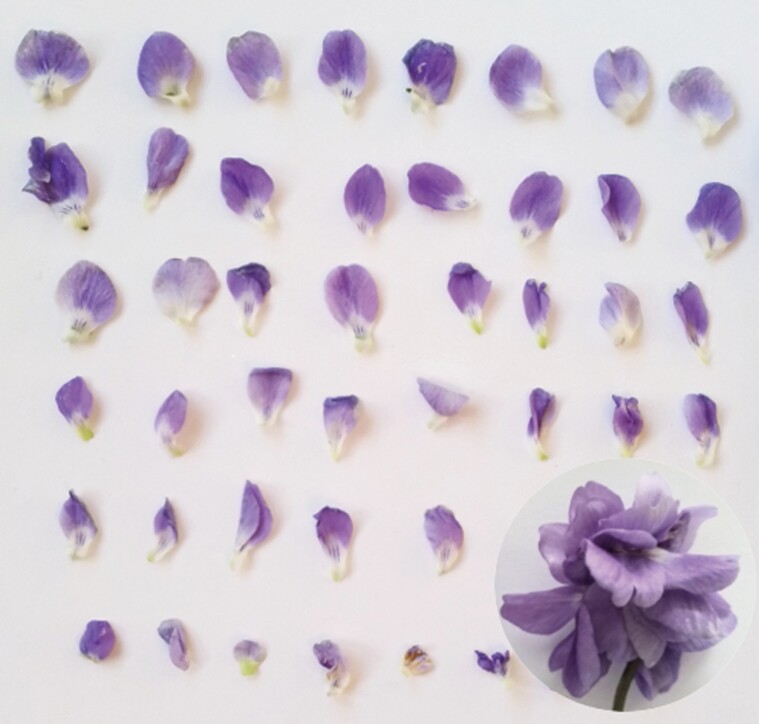
Chasmogamous flowers of *Viola alba* subsp. *dehnhardtii* harbouring 20–40 petals and petal-like stamens.

The *Viola* genus comprises some 500–600 species of violets and pansies whose fragrant and medicinal properties have long attracted botanists and herbalists. Among them, the ‘Parma violets’ are distinguished in several respects. While likely originating in Turkey, they were reported in Italy since the 16th century, notably in the area of Parma, hence their name, before being described in a few other places of Europe and North America in the 19th century (Mendonça de Carvalho *et al*. 2013). In the mid-19th century, they were brought to Toulouse (Southern France) where they became, and still are, the emblematic flower of the city, known ‘Toulouse violet’ (Morard *et al*. 2000). The uniqueness of this violet resides in its double, sweet-smelling chasmogamous flowers, which harbour 20–40 petals and petal-like stamens (Fig. 1). Accordingly, it is generally sterile, although easily propagated vegetatively.

The use of molecular phylogenetic techniques allowed Malécot *et al*. (2007) to rename the Parma ‘Violet of Toulouse’ as *Viola alba* subsp. *dehnhardtii.* More recently, its distinct taxonomic position was refined among a large collection of violets through genotypic and metabolomic analyses (Chervin *et al*. 2019). Despite being a valuable image for the phytomedicine and cosmetic industry, there was no chemical analysis of this violet until the recent report that leaf extracts of this plant are a promising source of antioxidant phenolic compounds (Chervin *et al*. 2017), consistent with extended studies on other *Viola* species (Vukics *et al*. 2008a, b; Koike *et al*. 2015; Moliner *et al*. 2019).

In the current study, we have taken advantage of the autofluorescence properties of phenolic compounds to gain a chemical imprint of their location *in situ*. This was achieved through a dual complementary approach that combines fluorescence microscopy imaging of living tissues of this violet, and chemical extraction of its main plant parts. Fluorescence imaging, on the one hand, is based on the natural emission of light in the visible spectrum (400–700 nm) of fluorescent cell compounds, most notably the phenolics C6–C3 hydroxycinnamic acids and coumarins, the more complex flavonoids and the highly polymerized lignin. Upon excitation of the samples using UV to red light, their fluorescence pattern can be observed through classical epifluorescence microscopy, and the corresponding emission spectra recorded by confocal microscopy (Hutzler *et al*. 1998; Buschmann *et al*. 2000; Talamond *et al*. 2015). Extraction protocols, on the other hand, can provide an insight into the chemical composition of plant extracts (Dai and Mumper 2010). In this study, the fluorescence of the flower, leaf and root tissues of the Parma violet were recorded, and their spectra compared to the spectra of the chemical compounds recovered upon extraction of the same organs. The data showed the feasibility to track the chemical profile of living tissues through fluorescence imaging, without the need of time-consuming, destructive biochemical techniques.

## Materials and Methods

### Plant material

Plants grown in green houses under controlled conditions were supplied by the dedicated facilities of the city of Toulouse (Southern France) at flowering time in early March over a period of 3 years (2017–2019). The flowers, leaves and roots were harvested separately, and either directly processed or lyophilized and stored at −20 °C until use.

### Plant extracts

Plant material was ground to a fine powder in liquid nitrogen with a mortar and pestle and then extracted following a protocol adapted from Craik *et al*. (1999). The powder was suspended in a solution of dichloromethane/methanol (v/v) at a ratio of 10 mL per gram fresh weight (FW) of the starting material. After stirring at room temperature for 4 h, the suspension was centrifuged (8000*g*, 10 min, 3 °C), and the pellet re-extracted for an additional 3 h using the same conditions. The pellet was discarded and the recovered supernatants were pooled, filtered on a sintered G5 glass funnel, then poured into a separating funnel and water added until a separation phase was visible. The lower phase which contains chlorophyll and lipophilic substances was discarded, and the upper methanolic aqueous phase was recovered, concentrated under reduced pressure (Büchi) and adjusted to a small volume with water, giving rise to the flower, leaf and root aqueous crude extracts (CEs).

### Biochemical assays

The quantitative determination of the phenolic and flavonoid contents of the CEs was achieved using standardized colourimetric methods: the Folin–Ciocalteu assay for measuring the C6–C3 phenolic compounds at 765 nm (Hughes *et al*. 2010) and the AlCl_3_ assay for measuring flavonoids at 430 nm (Bahorun *et al*. 1994). The total phenolic and flavonoid contents were calculated on the basis of the calibration curve of ferulic acid (*y* = 0.0168*x* + 0.0881, *R*^2^ = 0.9913) and rutin (*y* = 0.0211*x* + 0.0003, *R*^2^ = 0.9978), respectively. The anthocyanidin content was determined at 530 nm as reported by Ganjewala *et al*. (2008) with cyanidin chloride as the standard. The data were expressed as micrograms equivalent of ferulic acid, rutin or cyanidin chloride per gram of FW starting material.

### Ultra-high-performance liquid chromatography–high-resolution mass spectrometry profiling

Ultra-high-performance liquid chromatography–high-resolution mass spectrometry (UHPLC–HRMS) analysis of the phenolic compounds of flower, leaf or root extracts was performed as previously reported (Chervin *et al*. 2017) with a UHPLC-LTQ Orbitrap XL instrument (Ultimate 3000, Thermo Fisher Scientific, Hemel Hempstead, UK) and a Acquity UPLC BEH C18 column (100 × 2.1 mm i.d., 1.7 μm, Waters, MA, USA) equipped with a guard column. The mobile phase A (MPA) was water with 0.1 % formic acid (FA) and mobile phase B (MPB) was acetonitrile with 0.1 % FA. The solvent gradient was as follows: 0 min, 95 % MPA; 0.5 min, 95 % MPA; 12 min, 5 % MPA; 15 min, 5 % MPA; 15.5 min, 95 % MPA; and 19 min, 95 % MPA. The flow rate was 0.3 mL min^−1^, the column temperature was set to 40 °C and injection volume fixed to 2 μL. Mass detection was performed using an electrospray source (electrospray ionization technique) in negative ionization modes. The mass scanning range was *m/z* 100–2000 Da. Each full MS scan was followed by data-dependent tandem mass spectrometry (MS/MS) on the three most intense peaks.

Data files were processed with MS-DIAL version 3.08 (Tsugawa *et al*. 2015) with optimized detection threshold set to 3 × 10^5^. Annotation of main features was processed with MS-FINDER version 3.04 (Tsugawa *et al*. 2016) using an in-house *Viola* database based on matches within the Dictionary of Natural Products (DNP, CRC Press, version 25:2). Results afforded several candidates and they were ranked according to their similarity score based on comparison between experimental MS/MS fragments and *in silico* spectra of candidates.

### Microscopy

For optical microscopy, 150- to 200-µm sections of fresh samples were made using a vibratome (VT1000S, Leica, Rueil-Malmaison, France), mounted on a glass slide in a drop of distilled water and observed using either a fluorescent macroscope (AxioZoom V16, Zeiss, Germany) or an inverted microscope (DMIRBE, Leica, Germany) with a CCD camera (MC190HD, Leica, Germany). Plant fluorescent tissue imprints were observed with epifluorescence using different excitation ranges: UV (excitation filter BP 340–380 nm, emission filter LP 425 nm), blue (excitation filter BP 450–490 nm, emission filter LP 515 nm) and green (excitation filter BP 515–560 nm, emission filter LP 590 nm). Observations were made on 10 different leaves, roots and petals from two plants.

Confocal images were acquired with a confocal laser scanning microscope (CLSM; TCS SP8, Leica, Germany) using 405 and 561 nm diode lasers, with the emitted autofluorescence collected in the range of 420–600 and 650–720 nm, respectively. Images were acquired in the z dimension and 3D reconstructions were carried out using 3D Leica software. Emission spectra of intact cells and extracts were collected using the spectral module of the Leica SP2 scanning head. The 405-diode laser was used for excitation and spectra were collected in the range of 415–780 nm with a 10-nm band pass. Emission spectra (*N* = 20–30) were acquired for 10–15 cells per tissue types from two plants or from extracts made at least from three biological repeats.

## Results

### Visualization of autofluorescence within the different plant parts of *Viola*

**Figure 2.**
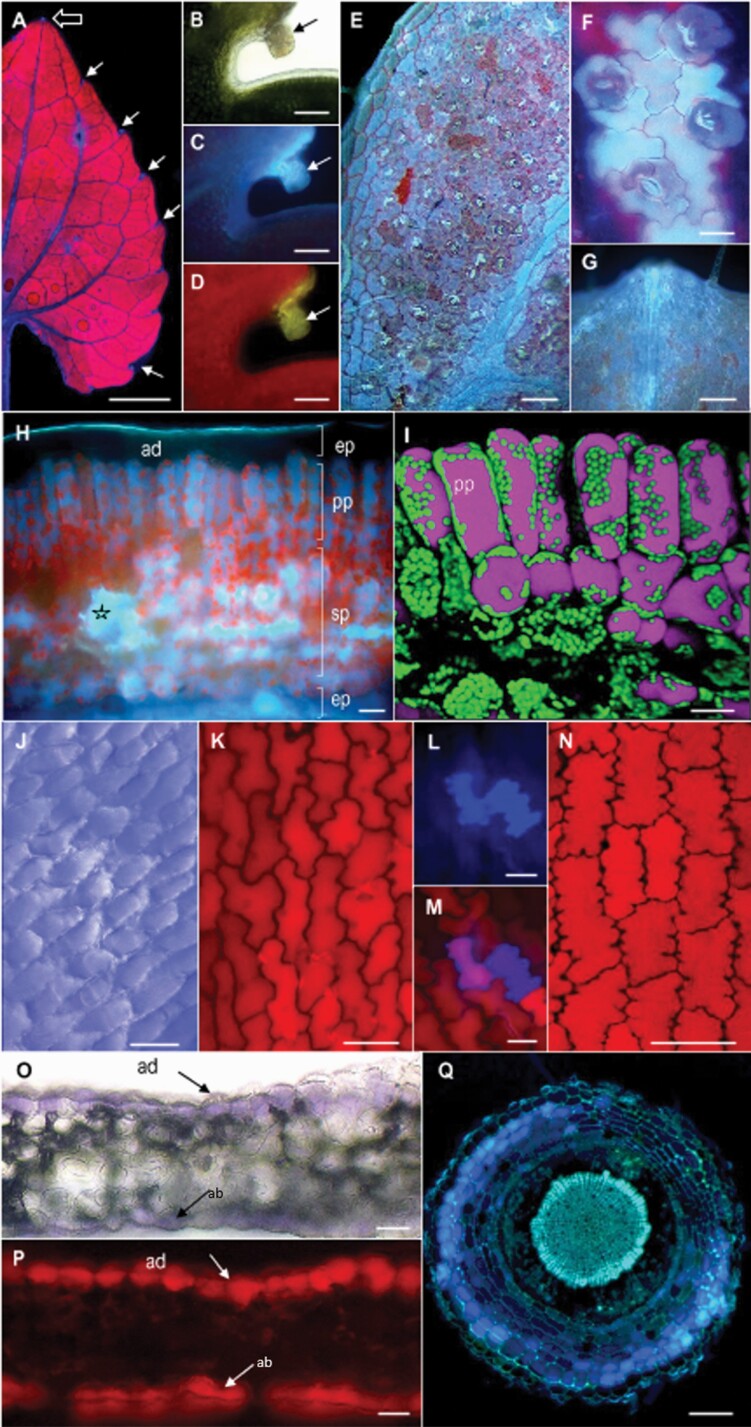
Overall views and transverse sections of leaf blade (A–I), petal (J–P) and root (Q). (A–I) Observation in epifluorescence wide-field microscopy of the adaxial face of the leaf blade excited at the UV range (A). Note the small blue spots at the end of the midrib (open arrow), and at each tooth of the dentate blade (arrows); the latter are shown at higher magnification in bright field (B) and fluorescence microscopy upon excitation in the UV range (C) and blue range (D), respectively. Scale bars: 1 mm (A), 100 µm (B–D). (E–G) Observation by epifluorescence wide-field microscopy of the abaxial face of the leaf blade excited in the UV range reveals the numerous stomata and blue patches of fluorescence (E); detail of the latter showing the epidermal cells surrounding the stomata strongly emitting in bright blue (F) and the area at the tip of the midrib (G). Scale bars: 200 µm (E), 50 µm (F), 400 µm (G). (H, I) Transverse sections of the leaf blade observed in wide-field (H) and confocal (I) fluorescence microscopy. Details from adaxial (ad) to abaxial (ab) face: cuticle of the epidermal cell layer, epidermis (ep), palisadic parenchyma (pp), spongy parenchyma (sp), pocket filled with fluorescent compounds (open star). Scale bars: 200 µm (H), 300 µm (I). (J–P) Observation of the adaxial (J–M) and abaxial (N) faces of a petal in bright field (J) and fluorescence microscopy (K–N), with a focus on cells which fluoresce both in the red (K) and blue range (L), and image overlay (M); transverse section of the petal (ad, adaxial epidermis) in bright field (O) and fluorescence (P) microscopy upon excitation at the UV range. Scale bars: 30 µm (L, M, O, P), 50 µm (J, K), 100 µm (N). (Q) Transverse section of the root showing the rhizoderm, xylem vessels and the pith. Scale bars: 300 µm.

In order to characterize the Parma violet *Viola alba* subsp. *dehnhardtii*, intact and/or transverse sections of the leaf blade, petal and root were examined (Fig. 2A–Q). Upon excitation in the UV range (340–380 nm), the fluorescence emitted (425 nm) by the adaxial (upper) surface of the leaf blade exhibited an intense fluorescence in the red range interspersed with the deep blue fluorescence of the midrib and the veins (Fig. 2A). This blue fluorescence was also detected at the distal part of the midrib (open arrow) and under the form of small outgrowths at each tooth of the dentate blade (solid arrows). Examined at higher magnification, these outgrowths resemble glandular structures in bright field (Fig. 2B), which emit blue (Fig. 2C) or yellow (Fig. 2D) fluorescence upon excitation in the UV (340*–*380 nm) or blue (450–490 nm) range.

Compared to the adaxial face, the abaxial (lower) surface exhibited a more intense fluorescence in the blue range than in red (Fig. 2E). The blue fluorescence was distributed in patches throughout the blade which holds numerous stomata, each being surrounded by highly fluorescent epidermal cells arranged in a typical jigsaw puzzle-type pattern (Fig. 2F); an intense fluorescence was also observed at the distal part of the midrib (Fig. 2G).

Observations of free-hand transverse sections of a leaf blade were made by conventional wide-field microscopy (Fig. 2H) and CLSM (Fig. 2I). Upon excitation in the UV range, the emitted fluorescence was observed mainly in the blue and the red channels, which roughly correspond to the fluorescence of phenolic compounds and chlorophyll, respectively. Blue fluorescence was associated primarily with the large vacuoles of palisade parenchyma cells (deep blue), large pockets within the spongy mesophyll (bright blue) and epidermal cells at the abaxial (lower) face of the leaf blade. Such a blue gradient might reflect the presence of distinct phenolic families. Cells of the adaxial epidermal layer covered by a thin fluorescent cuticle exhibited a distinct absence of fluorescence (Fig. 2H).

The red fluorescence clearly depicted the numerous chloroplasts dispersed in the cytosol around the vacuoles in leaf tissue. For a further examination, confocal images were acquired in the z dimension and used to generate 3D reconstructions focused on the mesophyll (Fig. 2I). As indicated above, the former range of emitted fluorescence (420–600 nm, pink pseudo colour) was mainly collected from the large vacuoles of both types of cells, and the latter (650–720 nm, green pseudo colour) from the numerous chloroplasts.

The general features of the flower petal are shown in Fig. 2J–P. Examination of intact petals and transverse sections showed that the violin colour was exclusively located in the epidermal layers, both on the adaxial and abaxial sides of the petal (Fig. 2O), typical of anthocyanidin pigments. This is consistent with the fluorescence properties of these two cell layers, which was mainly in the red range (Fig. 2P). A closer examination of the adaxial surface showed the jigsaw puzzle-like disposition of the cells (Fig. 2J and K), with a few cells exhibiting a blue autofluorescence (Fig. 2L and M). The cells of the abaxial surface exhibited different patterns, with the cell walls appearing to expand into the cellular space (Fig. 2N).

A transverse section of a *Viola* root is shown in Fig. 2Q. The emitted fluorescence was mainly collected in the blue range within cortical tissues, vascular tissues and the pith, again exhibiting a gradient from deep blue (two to three cell layers of the rhizoderm), to the bright turquoise blue of the xylem vessels and pith. This is indicative of the presence of different phenolics in these tissues, i.e. soluble phenolics in the vacuoles of the rhizoderm, versus insoluble wall-bound polymers in the vessels. Due to the high fluorescence intensity, the endodermis and pericycle tissues were not clearly delimited. Highly fluorescent patches, presumably secretory cells, were also visible at the outer cell layer of the rhizoderm.

### Chemical analysis of *Viola* tissue extracts

**Figure 3.**
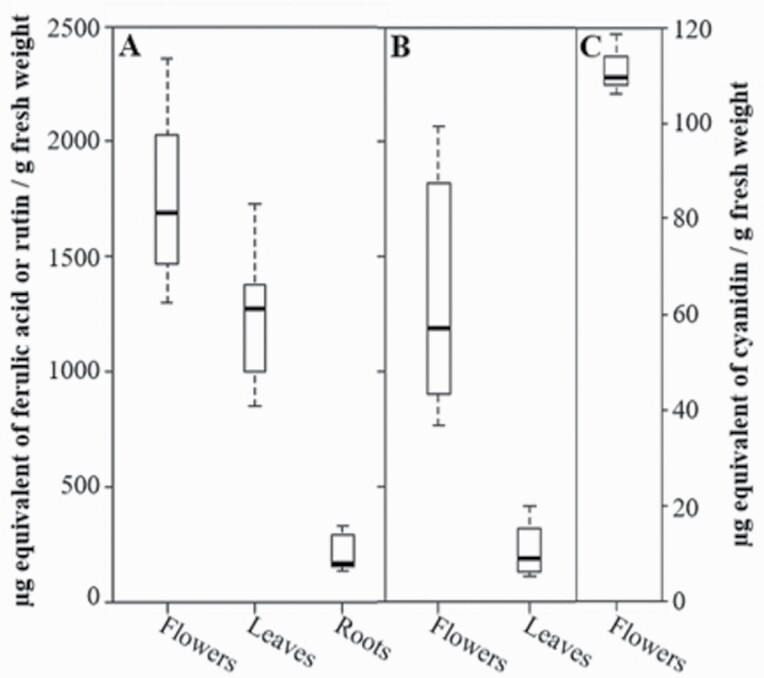
Phenolic acids (A), flavonoids (B) and anthocyanidins (C) content (µg g^−1^ FW) of flowers, leaves and root extracts. Whiskers represent max and min, the box edges are the first and third quartiles and the middle line represents the median of at least three independent biological repeats.

The protocol that was retained to solubilize small metabolites was adapted from Craik *et al*. (1999). Indeed, colourimetric assays showed that the aqueous CEs contain flavonoids and/or simple phenolics, whose amounts vary with the organs (Fig. 3). Over several extractions, the highest levels of flavonoids and phenolics characterized the flowers (mean values 1.34 ± 0.17 and 1.76 ± 0.11 mg g^−1^ FW, respectively), as compared to leaves (0.22 ± 0.03 and 1.26 ± 0.08 mg g^−1^ FW, respectively) and roots (0.2 ± 0.02 mg g^−1^ FW phenolics only). A further experiment conducted on a narrow (3–5 mm) band of tissue excised from the margin of the leaf blade revealed that the small outgrowths observed by microscopy (Fig. 2A–D) contributed significantly (up to 60–70 % per gram of FW) to the phenolic C6–C3 and flavonoid content of the leaf, being more concentrated in this part than in the rest of the blade.

Among phenolic compounds, preliminary UHPLC–HRMS analysis [**see****Supporting Information—Fig. S1** and **Table S3**] detected varying amounts of coumarins (peaks # 4, 8, 9) and phenolic acids, notably salicylic acid (peak # 44) in the aerial parts and root extracts of the violet, being higher in leaves than in other organs, on a FW basis. It also showed the richness of flower extracts in several flavonoids, most notably kaempferol (peaks # 16, 24, 26) and rutin glycosides (peaks # 46, 48). Anthocyanidins were restricted to flower extracts (Fig. 3), whose measured amounts (111.4 ± 3.2 µg g^−1^ FW) accounted for about 8 % of the total flavonoid content.

### Fluorescence emission spectra of intact tissues and tissue extracts

**Figure 4.**
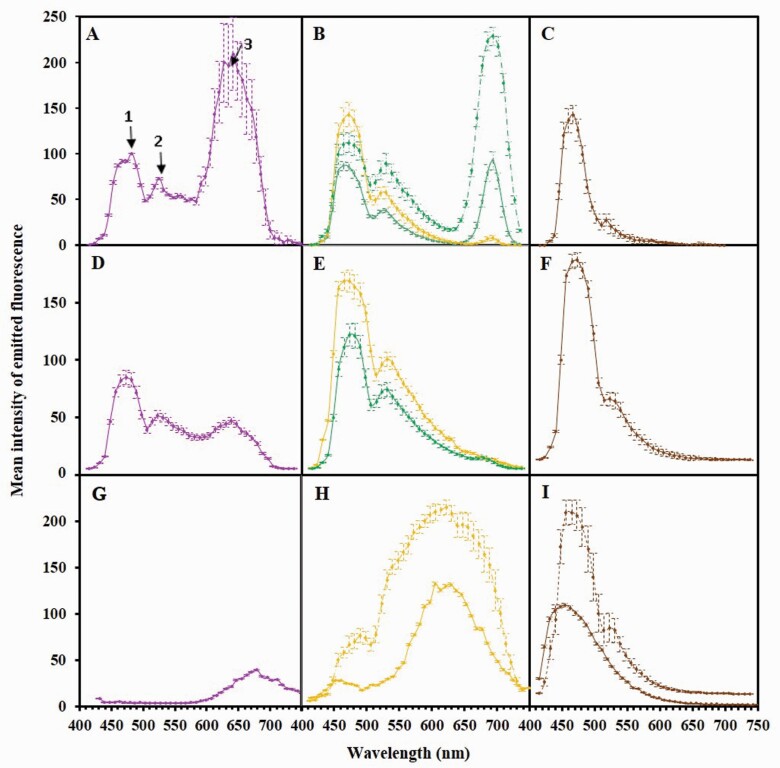
Fluorescence emission spectra (mean intensity in arbitrary units ± standard error vs. wavelength in nanometers) of living plant parts (A–C), extracts from the corresponding tissues (D–F) and reference compounds (G–I). (A–C) Spectra from: 15 petal epidermal cells (A); 20 different cells of palisadic parenchyma (dotted green), spongiform parenchyma (green) and glandular structure (yellow) (B); and 20 root cortical cells (C). (D–F) Spectra of extracts from 20 mg FW of: petals (D); leaves (green) and leaf borders (yellow) (E); and roots (F). (G–I) Spectra of reference compounds: the anthocyanidin-cyanidin chloride (G); the rutin (yellow) and quercetin (dotted yellow) flavonoids (H); the ferulic-hydroxycinnamic acid (brown); and the 4-methyl umbelliferone coumarin (dotted brown) (I).

To determine whether the compounds detected in the spectrophotometric assays (Fig. 3) could account for the autofluorescence observed in these tissues (Fig. 2), the fluorescent spectra of living tissues were determined using the lambda scan module of the CLSM. Using the 405-nm diode laser, spectra were collected from 415 to 780 nm, comparable to the conditions used for imaging, above. Figure 4 and Supporting Information—Table S2 show the spectra acquired for 15–20 cells per tissue types from two plants (Fig. 4A–C), the spectra of the aqueous extracts prepared from the same plant parts (Fig. 4D–F) and the spectra of standards (Fig. 4G–I) used for the chemical measurements above.

Based on the emission spectra, three distinct patterns were observed. The first of these (peak 1, *A*_max_ = 470 nm) was present in both intact tissues (Fig. 4A–C) and extracts (Fig. 4D–F) of flowers, leaves and roots. Because flavonoids were not detected in roots, it follows that peak 1 corresponds to the C6–C3 phenolic acid and/or coumarin compounds, in agreement with the spectra of the authentic standards (ferulic acid, 4-methyl umbelliferone). A closer examination of living leaf tissues showed that the fluorescence spectra displayed the same pattern across the section, being higher in the glandular-like structure and their extracts than in parenchyma cells (Fig. 4B and E), consistent with their respective concentration in C6–C3 compounds.

The second pattern, in the blue-green to yellow region of the visible spectrum (520–620 nm), was solely present in the leaf and flower tissues in the form of a peak (# 2) which slightly overlaps peak 1 in living tissues (Fig. 4A and B) and in the corresponding extracts (Fig. 4D and E). The presence of flavonoids in the extracts (Fig. 3B), including rutin [**see****Supporting Information—Fig. S1** and **Table S1**], and the region of the visible spectrum where these fluoresce, indicate that peak 2 corresponds to the flavonol group of flavonoids, consistent with the similar range of fluorescence of the flavonoid standards, rutin and quercetin (Fig. 3H).

A third emission maximum (peak 3, 600–700 nm) was specifically associated with the flower petal epidermis, both in the adaxial cells of intact tissues (Fig. 4A) and in extracts (Fig. 4D), typical of the presence of anthocyanidin flavonoids and in agreement with the spectrum of the authentic standard, cyanidin. While only observed in trace amounts in leaf extracts (Fig. 4E), its presence was likely also masked by the intense overlapping fluorescence of chlorophyll in these tissues (Fig. 4B).

## Discussion

In recent studies on various plants, CLSM has been used as a tool to characterize the autofluorescence of fresh tissues, and assess their content through their fluorescence emission spectra (Roshchina *et al*. 2017; Donaldson and Williams 2018; Mishra *et al*. 2018). The ability of phenolic compounds to autofluoresce upon illumination by UV or blue light (Hutzler *et al*. 1998; Buschmann *et al*. 2000; Talamond *et al*. 2015; Donaldson 2020) was utilized in order to explore the nature and location of these metabolites within different tissues of *Viola alba* subsp. *dehnhardtii* plants. The novelty of our study was to convert fluorescence imaging into a chemical profile by combining fluorescence microscopy and biochemical measurements. The chemical extracts needed for this purpose were prepared according to a protocol that solubilizes small metabolites, minimizes the extraction of high-molecular-weight compounds, mainly proteins and mucilaginous material, and allows to get rid of chlorophyll and lipophilic material (Craik *et al*. 1999). The recovered aqueous extracts, whose phenolic content was comparable to the values of other *Viola* species (Karioti *et al*. 2011; Koike *et al*. 2015; Benvenuti *et al*. 2016; Moliner *et al*. 2019), were used throughout this study. The retained approaches proved complementary in that non-invasive fluorescence microscopy anticipated the main classes of fluorophores in various living tissues, while biochemical analyses allowed for quantification, but not the precise localization, of the same classes of compounds. A major advance of this study was to integrate these two types of data by comparing their respective fluorescence spectra through CLSM.

A first outcome of this dual approach was to highlight novel and/or unusual cell type patterns. One of the distinctive features was the presence of glandular-like outgrowths at each indentation of the leaf blade. Autofluorescence in the blue-green to yellow region of the visible spectrum, indicative of the presence of phenolic acids, coumarins and flavonoids, was confirmed by the fluorescence spectra of extracts and intact tissues, and comparison to the phenolic compounds identified by HPLC-MS analysis of leaf extracts (Chervin *et al*. 2017) [**see****Supporting Information—Table S1**]. Although sharing the same phenolic profile as the whole blade, their cells differed from mesophyll tissues in the quasi absence of chlorophyll, rather appearing more as a reservoir of autofluorescent, possibly scent, highly concentrated secondary metabolites. Whether additional metabolites known to fluoresce in the region of the visible spectrum might be present (García-Plazaola *et al*. 2015) was not investigated. Interestingly, we observed these structures in all *Viola* species we have examined so far (*V*. *odorata*, *V. tricolor*, *V.* × *wittrockiana*, and many others); such outgrowths had not been reported in earlier studies on the foliar anatomy of this and other *Viola* species (Morard *et al*. 2000; Mehrvarz *et al*. 2013; Pilberg *et al*. 2016). A peculiar trait was also the phenolic acid richness of the cells surrounding the stomata, and the intense fluorescence at the distal part of the midrib.

An additional typical character of this violet, and of other *Viola* species, was the contrasted aspects of the petal adaxial and abaxial epidermis. Although equally rich in anthocyanidins and other flavonoids as revealed by fluorescence imaging and confirmed through their spectrum, the abaxial face was distinguished by a lace-like design which results from the shape of the cells, whose cell walls appear to regularly protrude inward into the cellular space. One may assume that such peculiar ingrowths, already observed in *Viola* × *wittrockiana* (Weryszko-Chmielewska and Sulborska 2012), reinforce both the strength and the exchange capabilities of the cell surface. Comparing the chemical composition of the cell walls of the two epidermis might enlighten their respective roles.

The most striking feature of roots was the intense blue colour observed in cross-sections, which is characteristic of the presence of C6–C3 phenolic compounds, most notably coumarins, as confirmed by comparison with authentic standards. This colouration was restricted to three cortical cell layers and was evenly distributed, apart from a few larger, even more intensely coloured cells, presumably corresponding to secretory cells that have been described in other *Viola* species (Hayden and Clough 1990). Whether phenolic acids might be associated with other compounds, such as the abundant mucilaginous material of root tissues, was not investigated.

A common trait to the three organs was the abundance of phenolics at strategic locations, i.e. on the leaf margin, around the stomata, within the flower epidermis and the root cortex. Due to their antioxidant activity, these compounds are known to protect plants against adverse conditions and exert antimicrobial activities (García-Plazaola *et al*. 2015). This suggests that they are part of the constitutive plant defence against possible invaders. Besides this role, it was recently reported that coumarins contribute to the plant fitness by shaping the root microbiome, and facilitating iron uptake from the soil environment (Tsai and Schmidt 2017; Stassen *et al*. 2020).

An additional noticeable, key finding of this study, was that the spectral signatures of endogenous fluorophores of intact violet tissues and the corresponding tissue extracts share the same fluorescence patterns. The fact that the respective peak heights were not proportional to the measured amounts of solubilized fluorophores likely results from the many factors that may affect fluorescence intensity such as pH, solvent polarity, chemical composition and quenching by pigments. Although this does not enable to quantify phenolics *in vivo* on the basis of spectral profiles, it confirms that one can certainly deduce their relative levels in cells or tissues of a given organ of this plant. Moreover, the use of non-destructive microscopy makes it possible to track the chemical profile of specific plant tissues in response to various environmental conditions without the need for time-consuming, destructive biochemical techniques (García-Plazaola *et al*. 2015). Such information might also aid in selecting optimal times or conditions for analyses involving more detailed chemical or metabolomic characterization.

A more precise location of the various metabolites would benefit from the powerful matrix-assisted laser desorption/ionization–mass spectrometric imaging technique, as illustrated on other plants (Ye *et al*. 2013).

## Conclusion

The dual chemical and imaging approach retained in this study provides a comprehensive histochemical localization of phenolics in living tissues of a violet. The advanced examination of the fluorescence imprint of *Viola alba* subsp. *dehnhardtii* not only enhances our knowledge of this plant but also paves the way for deciphering the potential of other *Viola* and plant species. It emphasizes that one can rely on autofluorescence of living tissues as a quality control of their richness in phenolic compounds, and as a marker of plant responses to environmental constraints. Together with the recent localization of defence peptides in another violet species (Slazak *et al*. 2018), it sheds light on the immune potential of violets to fight adverse conditions.

## Supporting Information

The following additional information is available in the online version of this article—

**Table S1.** Annotated phenolic compounds with MS-FINDER *in silico* matches in the crude extracts from 100 mg FW of flowers, leaves or roots of the Parma violet plants.

**Table S2.** Data of phenolic acids, flavonoids and anthocyanidins content (µg g^−1^ FW) of flowers, leaves and root extracts.

**Table S3.** Data of fluorescence emission spectra of living plant parts, extracts from the corresponding tissues and reference compounds.

**Figure S1.** UHPLC–HRMS/MS profiles in negative mode of the crude extracts from 100 mg FW of flowers (violet), leaves (green) or roots (red) of the Parma violet plants.

plab041_suppl_Supplementary_Figure_S1

plab041_suppl_Supplementary_Table_S1

plab041_suppl_Supplementary_Table_S2

plab041_suppl_Supplementary_Table_S3

## Data Availability

Data used for the chemical analysis and the fluorescence emission spectra are available as Supporting Information.

## Notes

**Phenome, Genome & Environment. Chief Editor:** Colleen Doherty
