# Supplementary material for: Phenolic profile of a Parma violet unveiled by chemical and fluorescence imaging
Source: AoB Plants. 2021 Jul 6;13(4):plab041. doi: 10.1093/aobpla/plab041 (PMC8300547; doi:10.1093/aobpla/plab041)

**Figure S1.** UHPLC-HRMS/MS profiles in negative mode of the crude extracts from 100 mg FW of flowers (violet), leaves (green) or roots (red) of the Parma violet plants.


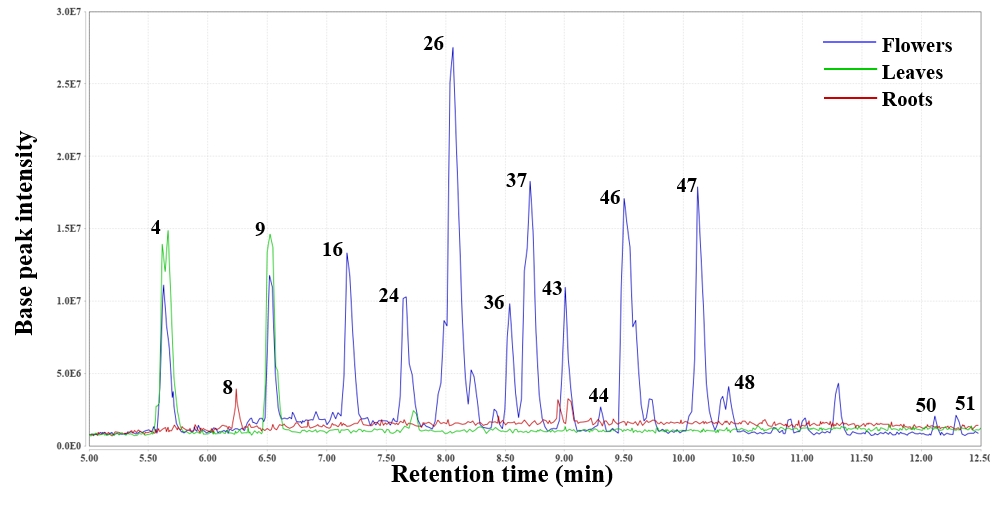

Supplement: plab041_suppl_Supplementary_Figure_S1 [file plab041_suppl_supplementary_figure_s1.docx]
