# Supplementary material for: Phenolic profile of a Parma violet unveiled by chemical and fluorescence imaging
Source: AoB Plants. 2021 Jul 6;13(4):plab041. doi: 10.1093/aobpla/plab041 (PMC8300547; doi:10.1093/aobpla/plab041)
Supplement: plab041_suppl_Supplementary_Table_S1 [file plab041_suppl_supplementary_table_s1.doc]

**Table S1.** Annotated phenolic compounds with MS-FINDER *in silico* matches in the crude extracts from 100 mg FW of flowers, leaves or roots of the Parma violet plants.

| ***Peak*** | ***Rt***  ***(min.)*** | ***[M-H]-*** | ***Chemical class*** | ***Molecular formula*** | ***Putative annotation*** | ***Normalized peak area*** | | | | | |
| --- | --- | --- | --- | --- | --- | --- | --- | --- | --- | --- | --- |
|  |  |  |  |  |  | ***Flowers*** | ***Leaves*** | | ***Roots*** | |  |
| **1** | 5.6 | 209.0451 | [Phenolic acids](http://phenol-explorer.eu/classifications/compounds/12) | C_10_H_10_O_5_ | 3-(2,4,5-Trihydroxyphenyl)-2-propenoic acid; 5-Me ether | 31893 | | 14544480 | | 9838 | |
| **2** | 5.62 | 595.1288 | Flavonoid glycosides | C_27_H_30_O_15_ | Kaempferol 7-glycosides; 7-*O*-[ β-D-Galactopyranosyl-(1→4)-α-L-rhamnopyranoside] | 12824 | | 4040428 | | 0 | |
| **3** | 5.64 | 769.1454 | Flavonoid glycosides | C_33_H_40_O_20_ | Kaempferol 3-glycosides; 3-*O*-[ α-L-Rhamnopyranosyl-(1→2)- β-D-glucopyranosyl-(1→6)-β-D-galactopyranoside] | 23695 | | 2264474 | | 0 | |
| **4** | 5.65 | 385.0767 | Coumarin glycosides | C_16_H_18_O_11_ | 5,6,7,8-Tetrahydroxy-2H-1-benzopyran-2-one; 7-Me ether, 5-*O*-β-D-glucopyranoside | 64387920 | | 96584360 | | 0 | |
| **5** | 5.66 | 221.0086 | Coumarin glycosides | C_10_H_6_O_6_ | 6,7-Dihydroxy-2-oxo-2H-1-benzopyran-3-carboxylic acid | 6041873 | | 10132060 | | 0 | |
| **6** | 5.95 | 327.1079 | [Phenolic glycosides](http://classyfire.wishartlab.com/tax_nodes/C0004165) | C_15_H_20_O_8_ | Salicin; 3'-Ac | 6109610 | | 116 | | 0 | |
| **7** | 6.11 | 299.0765 | [Hydroxybenzoic acid](http://phenol-explorer.eu/classifications/compounds/14/14) | C_13_H_16_O_8_ | 4-Hydroxybenzoic acid; *O*-β-D-Glucopyranoside | 4253104 | | 27689 | | 7944 | |
| **8** | 6.24 | 485.1291 | Coumarin glycosides | C_21_H_26_O_13_ | 7-*O*-β-Apiofuranosyl-(1→6)-β -glucopyranosylscopoletin | 22444 | | 2259317 | | 15087210 | |
| **9** | 6.53 | 385.0767 | Coumarin glycosides | C_16_H_18_O_11_ | 5,6,7,8-Tetrahydroxy-2H-1-benzopyran-2-one; 7-Me ether, 5-*O*-β-D-glucopyranoside | 54717030 | | 82727630 | | 0 | |
| **10** | 6.66 | 399.0925 | Phenolic acids | C_17_H_20_O_11_ | 2,6-Dihydroxybenzoic acid; 2-*O*-(2,3-Di-*O*-acetyl-β-D-glucopyranoside) | 18641 | | 2591170 | | 9465 | |
| **11** | 6.71 | 369.082 | Coumarin glycosides | C_16_H_18_O_10_ | 6,7,8-Trihydroxy-2H-1-benzopyran-2-one; 6-Me ether, 8-O-β-D-glucopyranoside | 2654345 | | 2301581 | | 0 | |
| **12** | 6.76 | 415.1601 | Coumarin glycosides | C_19_H_28_O_10_ | 1-(3,4-Dihydroxyphenyl)-5,6-dihydroxy-3-hexanone; 3'-Me ether, 4'-*O*-β-D-glucopyranoside | 0 | | 0 | | 7725176 | |
| **13** | 6.84 | 725.1924 | Flavonoid glycosides | C_32_H_38_O_19_ | Kaempferol 3-glycosides; 3-*O*-[β-D-Xylopyranosyl-(1→3)-α-L-rhamnopyranosyl-(1→6)-β-D-galactopyranoside] | 1902713 | | 18992 | | 0 | |
| **14** | 6.95 | 355.1027 | Phenolic acids | C_16_H_20_O_9_ | 3-(4-Hydroxy-3-methoxyphenyl)-2-propenoic acid; 4'-*O*-β-D-Allopyranoside | 4939104 | | 9076 | | 0 | |
| **15** | 7.06 | 593.1497 | Flavonoid glycosides | C_27_H_30_O_15_ | Kaempferol 3-glycosides; 3-*O*-[β-D-Glucopyranosyl-(1→2)-α-L-rhamnopyranoside] | 10475380 | | 4654006 | | 0 | |
| **16** | 7.18 | 901.2568 | Flavonoid glycosides | C_39_H_50_O_24_ | Kaempferol 3-[glucosyl-(1→3)-rhamnosyl-(1→2)-[rhamnosyl-(1→6)-galactoside]] | 39651 | | 0 | | 0 | |
| **17** | 7.22 | 593.1497 | Flavonoid glycosides | C_27_H_30_O_15_ | 8-C-β-D-Galactopyranosyl-4',5,7-trihydroxyflavone; 6''-*O*-β-D-Glucopyranosyl | 20498300 | | 2160343 | | 0 | |
| **18** | 7.31 | 771.1973 | Flavonoid glycosides | C_33_H_40_O_21_ | Kaempferol 3-glycosides; 3-*O*-[α-D-Glucopyranosyl-(1→4)-β-D-glucopyranosyl-(1→6)-β-D-glucopyranoside] | 12609320 | | 0 | | 0 | |
| **19** | 7.38 | 207.0295 | Coumarin glycosides | C_10_H_8_O_5_ | 6,8-Dihydroxy-3-(hydroxymethyl)-2H-1-benzopyran-2-one | 2295300 | | 17067 | | 0 | |
| **20** | 7.46 | 461.1655 | Tyrosols | C_20_H_30_O_12_ | 2-(4-Hydroxyphenyl) ethanol; Di-*O*-β-D-glucopyranoside | 2316073 | | 4345640 | | 2458860 | |
| **21** | 7.5 | 563.1391 | Flavonoid glycosides | C_26_H_28_O_14_ | 8-C-β-D-Glucopyranosyl-4',5,7-trihydroxyflavone; 2''-*O*-β-D-Xylopyranosyl | 3895553 | | 3775386 | | 0 | |
| **22** | 7.53 | 517.1549 | Hydroxybenzoic acid | C_22_H_30_O_14_ | 3,4,5-Trihydroxybenzoic acid; 3,5-Di-Me ether, Me ester, 4-*O*-[3-hydroxy-3-methylglutaroyl-(β6)-β-D-glucopyranoside] | 11872490 | | 423 | | 0 | |
| **23** | 7.61 | 625.1393 | Flavonoid glycosides | C_27_H_30_O_17_ | 3,4',5,7,8-Pentahydroxyflavone; 8-*O*-[β-D-Glucopyranosyl-(1→6)-β-D-glucopyranoside] | 5751080 | | 0 | | 0 | |
| **24** | 7.68 | 755.2022 | Flavonoid glycosides | C_33_H_40_O_20_ | Kaempferol 3-glycosides; 3-*O*- [α -L-Rhamnopyranosyl-(1→2)-α-D-glucopyranosyl-(1→6)-α-D-galactopyranoside] | 70853480 | | 4667 | | 0 | |
| **25** | 7.71 | 563.1391 | Flavonoid glycosides | C_26_H_28_O_14_ | 3,3',4',5,7-Pentahydroxy-6-methoxyflavone; 7-*O*-[2-Methylpropanoyl-(β6)-β-D-glucopyranoside] | 26379750 | | 16152240 | | 0 | |
| ***Peak*** | ***Rt***  ***(min.)*** | ***[M-H]-*** | ***Chemical class*** | ***Molecular formula*** | ***Putative annotation*** | ***Normalized peak area*** | | | | | |
|  |  |  |  |  |  | ***Flowers*** | | ***Leaves*** | | ***Roots*** | |
| **26** | 8.06 | 739.2075 | Flavonoid glycosides | C_33_H_40_O_19_ | Kaempferol 3-glycosides; 3-*O*-[α-L-Rhamnopyranosyl-(1→2)-α-L-rhamnopyranosyl-(1→6)-α-D-glucopyranoside] | 208153000 | | 29033 | | 0 | |
| **27** | 8.07 | 385.1131 | Phenolic acids | C_17_H_22_O_10_ | 3-(3,4,5-Trihydroxyphenyl)-2-propenoic acid; 3,5-Di-Me ether, 4-*O*-β-D-glucopyranoside | 16699 | | 3903748 | | 4120 | |
| **28** | 8.08 | 643.2221 | Phenolic acids | C_36_H_36_O_11_ | 4,8'-Oxylign-7-ene-3,3',4',7',9,9'-heptol; 7'-Et, 3,3'-di-Me ether, 9,9'-bis-*O*-(4-hydroxybenzoyl) | 0 | | 4414 | | 2846023 | |
| **29** | 8.21 | 609.1447 | Flavonoid glycosides | C_27_H_30_O_16_ | Quercetin 3-glycosides; 3-*O*-[β-D-glucopyranosyl-(1→4)-a-L-rhamnopyranoside] | 29000980 | | 794 | | 0 | |
| **30** | 8.23 | 465.1028 | Hydroxybenzyl aldehyde | C_21_H_22_O_12_ | 3,4-Dihydroxybenzaldehyde; 3-Me ether, 4-*O*-[3,4,5-trihydroxybenzoyl-(β6)-β-D-glucopyranoside] | 2615076 | | 0 | | 0 | |
| **31** | 8.24 | 341.1236 | Phenolic acids | C_16_H_22_O_8_ | 3-(3,4-Dihydroxyphenyl)-2-propen-1-ol; 3'-Me ether, 4'-*O*-β-D-glucopyranoside | 3112016 | | 0 | | 0 | |
| **32** | 8.29 | 533.1287 | Flavonoid glycosides | C_25_H_26_O_13_ | Quercetin 3-glycosides; 3-*O*-(6-*O*-Butyl-β-D-glucuronopyranoside) | 23574 | | 6733733 | | 0 | |
| **33** | 8.31 | 311.113 | Phenolic acids | C_15_H_20_O_7_ | 4-Hydroxyphenylacetic acid; Me ester, *O*-α-L-rhamnopyranoside | 7336677 | | 4514 | | 0 | |
| **34** | 8.41 | 463.0873 | Flavonoid glycosides | C_21_H_20_O_12_ | 3',4',5,5',7-Pentahydroxy-3-methoxyflavone; 3'-*O*-β-D-Xylopyranoside | 4168257 | | 0 | | 0 | |
| **35** | 8.42 | 593.1498 | Flavonoid glycosides | C_27_H_30_O_15_ | Kaempferol 7-glycosides; 7-*O*-[β-D-Galactopyranosyl-(1→4)-a-L-rhamnopyranoside] | 15097310 | | 1301 | | 0 | |
| **36** | 8.54 | 771.2336 | Flavonoid glycosides | C_34_H_44_O_20_ | 5,7,3'-Trihydroxy-4'-methoxyflavanone 7-galactosyl-(1→2)-[rhamnosyl-(1→6)] glucoside | 18526830 | | 0 | | 0 | |
| **37** | 8.55 | 341.1236 | Flavonoid glycosides | C_16_H_22_O_8_ | 3-(4-Hydroxyphenyl) propanoic acid; Me ester, *O*-β-D-glucopyranoside | 49463140 | | 0 | | 0 | |
| **38** | 8.71 | 449.2018 | Flavonoid glycosides | C_27_H_30_O_6_ | 3',4',5,7-Tetrahydroxyisoflavone; 3',4'-Bis(3-methyl-2-butenyl), 5,7-di-Me ether | 122341500 | | 0 | | 0 | |
| **39** | 8.78 | 593.1498 | Flavonoid glycosides | C_27_H_30_O_15_ | 3',4',5,7-Tetrahydroxyflavone; 4'-*O*-[α-L-Rhamnopyranosyl-(1→6)-β-D-glucopyranoside] | 14685210 | | 233 | | 0 | |
| **40** | 8.79 | 594.1525 | Flavonoid glycosides | C_27_H_30_O_15_ | Kaempferol 7-glycosides; 7-*O*-[β-D-Galactopyranosyl-(1→4)-α-L-rhamnopyranoside] | 4289000 | | 0 | | 0 | |
| **41** | 8.94 | 641.2068 | Catechol | C_29_H_38_O_16_ | 1-(3,4-Dihydroxyphenyl)-1,2-ethanediol; 3'-Me ether, 4'-*O*-[3,4-dimethoxybenzoyl-(β5)-β-D-apiofuranosyl-(1→6)-β-D-glucopyranoside] | 999 | | 0 | | 4863390 | |
| **42** | 8.98 | 447.0921 | Flavonoid glycosides | C_21_H_20_O_11_ | 6-C-β-D-Glucopyranosyl-3',4',7,8-tetrahydroxyflavone; 2''-*O*-α-L-Rhamnopyranosyl | 11051440 | | 459 | | 0 | |
| **43** | 9.01 | 405.1756 | Flavonoid glycosides | C_25_H_26_O_5_ | 5,7,4'-Trihydroxy-3',5'-diprenylisoflavone | 47540950 | | 0 | | 0 | |
| **44** | 9.17 | 137.0243 | Phenolic acids | C_7_H_6_O_3_ | 2-Hydroxybenzoic acid | 3964717 | | 6485640 | | 3127114 | |
| **45** | 9.51 | 475.1811 | Phenolic acids | C_21_H_32_O_12_ | 3-(3,4-Dihydroxyphenyl)-1-propanol; 3'-Me ether, 4'-*O*-[β-D-apiofuranosyl-(1→6)-β-D-glucopyranoside] | 6010436 | | 0 | | 0 | |
| **46** | 9.54 | 429.1758 | Flavonoid glycosides | C_20_H_30_O_10_ | Phenethyl rutinoside | 145619400 | | 0 | | 0 | |
| **47** | 10.14 | 431.1913 | Flavonoid glycosides | C_27_H_28_O_5_ | 5,7-dimethoxy-2-(4-methoxyphenyl)-4-[2-(4-methoxyphenyl) ethenyl]-3,4-dihydro-2H-1-benzopyran | 88630180 | | 0 | | 0 | |
| **48** | 10.39 | 429.1757 | Flavonoid glycosides | C_20_H_30_O_10_ | Phenethyl rutinoside | 20404130 | | 0 | | 0 | |
| **49** | 11.29 | 457.2068 | Unknown | C_22_H_34_O_10_ |  | 8012102 | | 0 | | 0 | |
| **50** | 12.12 | 475.1236 | Flavonoid glycosides | C_23_H_24_O_11_ | 8-C-β-D-Glucopyranosyl-4',5,6,7-tetrahydroxyflavone ; 6,7-Di-Me ether | 3132433 | | 0 | | 0 | |
| **51** | 12.27 | 505.1346 | Flavonoid glycosides | C_24_H_26_O_12_ | 7-(β-D-Glucopyranosyloxy)-5-hydroxy-6,8-dimethoxy-2-(4-methoxyphenyl)-4H-1-benzopyran-4-one | 33824 | | 0 | | 0 | |
