# Supplementary material for: Phenolic profile of a Parma violet unveiled by chemical and fluorescence imaging
Source: AoB Plants. 2021 Jul 6;13(4):plab041. doi: 10.1093/aobpla/plab041 (PMC8300547; doi:10.1093/aobpla/plab041)
Supplement: plab041_suppl_Supplementary_Table_S2 [file plab041_suppl_supplementary_table_s2.doc]

**Table S2. Data of phenolic acids, flavonoids and anthocyanidins content (µg.g^-1^ FW) of flowers, leaves, and root extracts.**

| **Crude Extract** | **Phenolic acids** | | | **Flavonoids** | | **Anthocyanidins** |
| --- | --- | --- | --- | --- | --- | --- |
|  | **µg equivalent of ferulic acid / g FW** | | | **µg equivalent of rutin / g FW** | | **µg equivalent of Cyanidin / g FW** |
|  | **Flowers** | **Leaves** | **Roots** | **Flowers** | **Leaves** | **Flowers** |
| **1** | 1900 | 1245 | 165 | 1580 | 330 | 118.55 |
| **2** | 1687 | 1356 | 122 | 1153 | 153 | 106.15 |
| **3** | 2360 | 937 | 326 | 2062 | 183 | 109.58 |
| **4** | 1628 | 1001 | 236 | 828 | 319 |  |
| **5** | 1529 | 1275 | 154 | 1003 | 108 |  |
| **6** | 1400 | 1000 | 152 | 1227 | 126 |  |
| **7** | 1894 | 1300 | 133 | 1897 | 194 |  |
| **8** | 2150 | 1433 | 310 | 2064 | 108 |  |
| **9** | 2153 | 1730 | 314 | 870 | 414 |  |
| **10** | 1371 | 1729 | 149 | 764 |  |  |
| **11** | 1300 | 852 | 165 |  |  |  |
| **12** |  | 1275 |  |  |  |  |
| **Average** | 1761 | 1260 | 202 | 1345 | 215 | 111.43 |
| **SE** | 107.57 | 86 | 23.79 | 162.93 | 37.22 | 3.7 |
| **Min** | 1300 | 852 | 122 | 764 | 108 | 106 |
| **Quart 1** | 1465 | 1001 | 151 | 903 | 126 | 108 |
| **Median** | 1900 | 1245 | 165 | 1153 | 183 | 110 |
| **Quart 3** | 2025 | 1375 | 273 | 1818 | 319 | 114 |
| **Max** | 2360 | 1730 | 326 | 2064 | 414 | 119 |
