# Supplementary material for: Phenolic profile of a Parma violet unveiled by chemical and fluorescence imaging
Source: AoB Plants. 2021 Jul 6;13(4):plab041. doi: 10.1093/aobpla/plab041 (PMC8300547; doi:10.1093/aobpla/plab041)
Supplement: plab041_suppl_Supplementary_Table_S3 [file plab041_suppl_supplementary_table_s3.doc]

Table S3. Data of fluorescence emission spectra of living plant parts, extracts from the corresponding tissues and reference compounds.

| **Petal epidermal cells** | | | | | | | | | | | | | | | |
| --- | --- | --- | --- | --- | --- | --- | --- | --- | --- | --- | --- | --- | --- | --- | --- |
|  | **Intensity of emitted fluorescence** | | | | | | | | | | | | | |  |
| **Wavelength nm** | **1** | **2** | **3** | **4** | **5** | **6** | **7** | **8** | **9** | **10** | **11** | **12** | **Average** | **SE** | |
| **415** | 0.116 | 0.820 | 0.820 | 0.116 | 0.116 | 0.116 | 0.053 | 0.053 | 0.053 | 0.641 | 0.641 | 0.451 | **0.333** | **0.091** | |
| **423** | 2.443 | 1.809 | 1.809 | 2.443 | 2.443 | 2.443 | 0.741 | 0.741 | 0.741 | 1.227 | 1.227 | 2.115 | **1.682** | **0.206** | |
| **431** | 7.649 | 7.870 | 7.870 | 7.649 | 7.649 | 7.649 | 6.482 | 6.482 | 6.482 | 3.574 | 3.574 | 9.598 | **6.877** | **0.508** | |
| **440** | 13.376 | 10.161 | 10.161 | 13.376 | 13.376 | 13.376 | 8.243 | 8.243 | 8.243 | 13.274 | 13.274 | 4.449 | **10.796** | **0.868** | |
| **448** | 36.175 | 32.992 | 32.992 | 36.175 | 36.175 | 36.175 | 26.370 | 26.370 | 26.370 | 37.707 | 37.707 | 25.546 | **32.563** | **1.430** | |
| **456** | 74.039 | 74.670 | 74.670 | 74.039 | 74.039 | 74.039 | 66.078 | 66.078 | 66.078 | 58.243 | 58.243 | 60.493 | **68.392** | **1.928** | |
| **464** | 93.995 | 101.232 | 101.232 | 93.995 | 93.995 | 93.995 | 92.135 | 92.135 | 92.135 | 79.661 | 79.661 | 87.643 | **91.818** | **1.965** | |
| **472** | 96.657 | 91.136 | 91.136 | 96.657 | 96.657 | 96.657 | 89.702 | 89.702 | 89.702 | 87.298 | 87.298 | 88.443 | **91.754** | **1.102** | |
| **481** | 105.356 | 99.338 | 99.338 | 105.356 | 105.356 | 105.356 | 97.775 | 97.775 | 97.775 | 95.155 | 95.155 | 96.403 | **100.012** | **1.201** | |
| **489** | 96.708 | 72.772 | 72.772 | 96.708 | 96.708 | 96.708 | 78.207 | 78.207 | 78.207 | 88.747 | 88.747 | 93.158 | **86.471** | **2.826** | |
| **497** | 69.806 | 73.601 | 73.601 | 69.806 | 69.806 | 69.806 | 63.417 | 63.417 | 63.417 | 55.981 | 55.981 | 58.956 | **65.633** | **1.830** | |
| **505** | 48.709 | 54.681 | 54.681 | 48.709 | 48.709 | 48.709 | 45.673 | 45.673 | 45.673 | 49.044 | 49.044 | 43.598 | **48.575** | **0.974** | |
| **513** | 57.773 | 50.179 | 50.179 | 57.773 | 57.773 | 57.773 | 43.638 | 43.638 | 43.638 | 58.292 | 58.292 | 54.108 | **52.755** | **1.798** | |
| **522** | 71.939 | 62.948 | 62.948 | 71.939 | 71.939 | 71.939 | 49.270 | 49.270 | 49.270 | 71.745 | 71.745 | 62.649 | **63.967** | **2.789** | |
| **530** | 61.804 | 48.584 | 48.584 | 61.804 | 61.804 | 61.804 | 58.429 | 58.429 | 58.429 | 68.228 | 68.228 | 66.613 | **60.228** | **1.866** | |
| **538** | 53.762 | 57.239 | 57.239 | 53.762 | 53.762 | 53.762 | 50.812 | 50.812 | 50.812 | 56.420 | 56.420 | 63.700 | **54.875** | **1.060** | |
| **546** | 53.487 | 50.879 | 50.879 | 53.487 | 53.487 | 53.487 | 44.456 | 44.456 | 44.456 | 55.883 | 55.883 | 62.059 | **51.908** | **1.541** | |
| **554** | 63.462 | 41.876 | 41.876 | 63.462 | 63.462 | 63.462 | 44.961 | 44.961 | 44.961 | 57.087 | 57.087 | 57.021 | **53.640** | **2.646** | |
| **563** | 47.914 | 48.162 | 48.162 | 47.914 | 47.914 | 47.914 | 62.233 | 62.233 | 62.233 | 52.061 | 52.061 | 54.438 | **52.770** | **1.761** | |
| **571** | 43.638 | 52.226 | 52.226 | 43.638 | 43.638 | 43.638 | 49.070 | 49.070 | 49.070 | 51.658 | 51.658 | 52.739 | **48.522** | **1.105** | |
| **579** | 45.251 | 49.667 | 49.667 | 45.251 | 45.251 | 45.251 | 51.593 | 51.593 | 51.593 | 56.655 | 56.655 | 51.549 | **49.998** | **1.197** | |
| **587** | 42.626 | 49.836 | 49.836 | 42.626 | 42.626 | 42.626 | 115.449 | 115.449 | 115.449 | 55.213 | 55.213 | 76.037 | **66.916** | **8.862** | |
| **596** | 40.804 | 82.823 | 82.823 | 40.804 | 40.804 | 40.804 | 128.925 | 128.925 | 128.925 | 47.307 | 47.307 | 94.222 | **75.373** | **10.810** | |
| **604** | 43.818 | 97.903 | 97.903 | 43.818 | 43.818 | 43.818 | 173.390 | 173.390 | 173.390 | 68.061 | 68.061 | 184.092 | **100.955** | **16.943** | |
| **612** | 52.437 | 123.283 | 123.283 | 52.437 | 52.437 | 52.437 | 274.822 | 274.822 | 274.822 | 76.298 | 76.298 | 283.670 | **143.087** | **29.439** | |
| **620** | 51.589 | 174.068 | 174.068 | 51.589 | 51.589 | 51.589 | 306.461 | 306.461 | 306.461 | 83.876 | 83.876 | 364.273 | **167.158** | **35.298** | |
| **628** | 52.121 | 218.613 | 218.613 | 52.121 | 52.121 | 52.121 | 376.108 | 376.108 | 376.108 | 86.982 | 86.982 | 446.406 | **199.534** | **45.014** | |
| **637** | 62.327 | 163.907 | 163.907 | 62.327 | 62.327 | 62.327 | 373.076 | 373.076 | 373.076 | 95.167 | 95.167 | 470.346 | **196.419** | **44.654** | |
| **645** | 63.113 | 202.489 | 202.489 | 63.113 | 63.113 | 63.113 | 395.630 | 395.630 | 395.630 | 84.425 | 84.425 | 495.239 | **209.034** | **47.886** | |
| **653** | 45.967 | 138.890 | 138.890 | 45.967 | 45.967 | 45.967 | 368.475 | 368.475 | 368.475 | 82.759 | 82.759 | 433.468 | **180.505** | **44.803** | |
| **661** | 48.553 | 152.663 | 152.663 | 48.553 | 48.553 | 48.553 | 293.787 | 293.787 | 293.787 | 75.498 | 75.498 | 385.693 | **159.799** | **35.750** | |
| **669** | 44.042 | 137.260 | 137.260 | 44.042 | 44.042 | 44.042 | 291.378 | 291.378 | 291.378 | 64.026 | 64.026 | 325.766 | **148.220** | **33.804** | |
| **678** | 33.859 | 84.930 | 84.930 | 33.859 | 33.859 | 33.859 | 250.385 | 250.385 | 250.385 | 42.078 | 42.078 | 280.515 | **118.427** | **30.279** | |
| **686** | 24.917 | 45.876 | 45.876 | 24.917 | 24.917 | 24.917 | 157.749 | 157.749 | 157.749 | 34.690 | 34.690 | 201.208 | **77.938** | **19.723** | |
| **694** | 15.440 | 29.678 | 29.678 | 15.440 | 15.440 | 15.440 | 83.155 | 83.155 | 83.155 | 18.788 | 18.788 | 87.594 | **41.313** | **9.276** | |
| **702** | 7.749 | 10.852 | 10.852 | 7.749 | 7.749 | 7.749 | 32.022 | 32.022 | 32.022 | 7.422 | 7.422 | 35.993 | **16.634** | **3.522** | |
| **710** | 3.522 | 4.933 | 4.933 | 3.522 | 3.522 | 3.522 | 14.555 | 14.555 | 14.555 | 3.374 | 3.374 | 16.361 | **7.561** | **1.601** | |
| **719** | 1.761 | 2.466 | 2.466 | 1.761 | 1.761 | 1.761 | 7.278 | 7.278 | 7.278 | 1.687 | 1.687 | 8.180 | **3.780** | **0.801** | |
| **727** | 2.289 | 3.206 | 3.206 | 2.289 | 2.289 | 2.289 | 9.461 | 9.461 | 9.461 | 2.193 | 2.193 | 10.634 | **4.914** | **1.041** | |
| **735** | 1.635 | 2.290 | 2.290 | 1.635 | 1.635 | 1.635 | 6.758 | 6.758 | 6.758 | 1.566 | 1.566 | 7.596 | **3.510** | **0.743** | |

| **Leaf palisadic parenchyma cells** | | | | | | | | | | | | | | | | | | | |
| --- | --- | --- | --- | --- | --- | --- | --- | --- | --- | --- | --- | --- | --- | --- | --- | --- | --- | --- | --- |
|  | **Intensity of emitted fluorescence** | | | | | | | | | | | | | | | | | | |
| **Wavelength nm** | **1** | **2** | **3** | **4** | **5** | **6** | **7** | **8** | **9** | **10** | **11** | **12** | **13** | **14** | **15** | **16** | **17** | **Average** | **SE** |
| **415** | 0.199 | 0.419 | 0.144 | 0.173 | 0.551 | 0.207 | 0.334 | 0.317 | 0.124 | 0.448 | 0.123 | 0.338 | 0.508 | 0.308 | 0.168 | 0.81 | 0.567 | **0.338** | **0.05** |
| **423** | 4.113 | 1.828 | 0.54 | 1.309 | 1.2 | 1.826 | 1.149 | 2.146 | 1.62 | 1.876 | 1.099 | 0.701 | 1.172 | 1.096 | 0.579 | 0.457 | 0.304 | **1.354** | **0.22** |
| **432** | 19.733 | 12.241 | 11.899 | 8.48 | 6.498 | 9.588 | 2.604 | 17.797 | 13.977 | 14.569 | 11.555 | 4.95 | 11.489 | 8.08 | 3.597 | 4.3 | 4.567 | **9.760** | **1.22** |
| **440** | 31.916 | 20.22 | 13.786 | 9.677 | 11.317 | 14.762 | 6.282 | 24.998 | 19.622 | 27.226 | 25.599 | 6.144 | 14.673 | 11.639 | 6.506 | 4.824 | 11.953 | **15.361** | **2.00** |
| **448** | 109.9 | 76.451 | 56.238 | 37.957 | 47.103 | 53.025 | 20.94 | 86.149 | 69.459 | 86.832 | 77.04 | 23.92 | 47.521 | 37.609 | 22.306 | 19.831 | 35.252 | **53.384** | **6.54** |
| **456** | 184.47 | 138.72 | 110.72 | 70.208 | 91.111 | 108.05 | 39.115 | 159.42 | 128.74 | 149.37 | 149.34 | 50.665 | 87.016 | 73.377 | 41.777 | 39.979 | 68.523 | **99.447** | **11.18** |
| **465** | 195.07 | 151.14 | 125.86 | 82.904 | 113.89 | 120.94 | 49.387 | 162.84 | 141.49 | 162.73 | 151.36 | 56.643 | 93.497 | 79.597 | 50.046 | 49.337 | 79.731 | **109.792** | **11.22** |
| **473** | 194.14 | 147.75 | 126.06 | 87.049 | 122.61 | 122.07 | 51.059 | 160.96 | 139.37 | 165.86 | 153.93 | 62.895 | 91.063 | 89.473 | 52.035 | 46.36 | 88.541 | **111.837** | **10.91** |
| **481** | 189.96 | 151.29 | 124.58 | 92.976 | 114.56 | 114.55 | 47.609 | 156.81 | 131.16 | 160.7 | 155.81 | 61.783 | 85.252 | 86.496 | 52.37 | 41.418 | 87.46 | **109.105** | **10.82** |
| **490** | 179.48 | 134.78 | 120.18 | 83.559 | 109.03 | 100.56 | 44.944 | 154.6 | 130.36 | 154.38 | 138.19 | 60.014 | 84.285 | 84.151 | 49.645 | 44.133 | 80.382 | **103.098** | **10.08** |
| **498** | 157.31 | 125.74 | 93.069 | 65.446 | 87.866 | 88.961 | 28.569 | 128.63 | 113.67 | 122.06 | 116.66 | 46.593 | 59.055 | 62.462 | 40.058 | 39.009 | 61.854 | **84.530** | **9.19** |
| **506** | 130.45 | 100.81 | 72.496 | 43.911 | 59.805 | 74.644 | 21.883 | 106.49 | 100.66 | 107.34 | 85.952 | 34.589 | 48.374 | 42.379 | 25.356 | 28.226 | 44.054 | **66.319** | **8.21** |
| **514** | 139.06 | 111.01 | 68.037 | 44.179 | 55.139 | 81.815 | 21.192 | 113.7 | 106.6 | 106.04 | 90.023 | 33.793 | 46.975 | 45.308 | 25.192 | 31.122 | 48.557 | **68.691** | **8.83** |
| **523** | 156.57 | 126.17 | 95.465 | 50.625 | 73.91 | 103.26 | 24.469 | 134.19 | 122.61 | 129.83 | 109.54 | 39.759 | 62.821 | 54.429 | 28.146 | 28.974 | 51.218 | **81.882** | **10.41** |
| **531** | 168.99 | 141.71 | 95.949 | 59.412 | 80.835 | 114.32 | 26.482 | 139.38 | 138.61 | 136.43 | 116.15 | 42.261 | 58.636 | 67.236 | 34.118 | 35.302 | 59.486 | **89.136** | **11.02** |
| **539** | 151.89 | 126.53 | 92.371 | 51.955 | 61.459 | 92.299 | 22.958 | 130.88 | 124.24 | 117.37 | 98.582 | 40.2 | 53.371 | 58.717 | 29.401 | 28.538 | 53.653 | **78.495** | **9.95** |
| **548** | 137.83 | 107.11 | 79.54 | 50.277 | 61.587 | 81.565 | 22.283 | 114.3 | 120.4 | 103.63 | 84.805 | 31.785 | 49.508 | 47.949 | 25.549 | 30.785 | 53.239 | **70.714** | **8.78** |
| **556** | 120.65 | 94.286 | 66.993 | 36.13 | 52.573 | 72.452 | 16.994 | 95.089 | 97.124 | 96.184 | 71.075 | 30.952 | 39.361 | 43.588 | 22.432 | 25.586 | 41.679 | **60.185** | **7.68** |
| **564** | 103.54 | 87.031 | 64.547 | 34.365 | 45.902 | 69.567 | 18.929 | 89.878 | 92.245 | 87.028 | 62.257 | 24.119 | 35.304 | 43.098 | 22.81 | 26.623 | 38.283 | **55.619** | **6.85** |
| **572** | 100.07 | 75.856 | 46.924 | 31.214 | 44.114 | 57.501 | 13.181 | 72.844 | 77.944 | 67.426 | 57.766 | 23.009 | 28.565 | 35.016 | 20.507 | 21.799 | 38.21 | **47.762** | **6.00** |
| **581** | 77.856 | 64.916 | 42.484 | 24.581 | 39.192 | 40.227 | 12.256 | 62.695 | 68.634 | 59.026 | 42.542 | 19.519 | 27.09 | 29.65 | 15.302 | 13.327 | 32.658 | **39.527** | **5.01** |
| **589** | 66.203 | 50.646 | 41.188 | 23.418 | 32.164 | 38.976 | 9.349 | 51.462 | 58.418 | 43.593 | 35.825 | 14.553 | 18.896 | 26.393 | 15.708 | 12.787 | 27.817 | **33.376** | **4.11** |
| **597** | 56.045 | 45.59 | 35.572 | 22.768 | 27.334 | 32.598 | 9.206 | 44.213 | 49.052 | 37.138 | 30.125 | 11.781 | 17.896 | 20.422 | 11.226 | 12.667 | 25.286 | **28.760** | **3.48** |
| **605** | 51.386 | 36.475 | 25.748 | 17.154 | 18.309 | 30.268 | 7.792 | 39.956 | 46.914 | 31.553 | 24.257 | 10.257 | 14.076 | 19.032 | 11.618 | 12.009 | 22.645 | **24.673** | **3.17** |
| **614** | 44.652 | 30.244 | 22.572 | 13.023 | 19.451 | 22.117 | 7.824 | 28.98 | 32.818 | 28.189 | 22.727 | 8.591 | 12.667 | 16.77 | 10.279 | 7.628 | 19.882 | **20.495** | **2.48** |
| **622** | 36.476 | 28.055 | 20.584 | 10.151 | 18.596 | 19.01 | 6.68 | 25.793 | 27.981 | 23.048 | 18.15 | 9.246 | 12.009 | 14.333 | 9.629 | 8.974 | 17.031 | **17.985** | **2.02** |
| **630** | 31.974 | 28.709 | 17.679 | 12.2 | 11.203 | 19.368 | 6.606 | 24.677 | 24.779 | 18.657 | 17.742 | 10.114 | 12.17 | 13.356 | 10.874 | 10.258 | 15.593 | **16.821** | **1.74** |
| **639** | 31.912 | 22.839 | 18.911 | 12.976 | 10.345 | 19.583 | 6.867 | 22.961 | 24.56 | 23.259 | 21.049 | 11.966 | 12.975 | 18.859 | 11.765 | 11.946 | 15.205 | **17.528** | **1.57** |
| **647** | 35.963 | 38.503 | 35.213 | 19.972 | 15.966 | 32.375 | 12.33 | 31.969 | 38.588 | 27.931 | 26.001 | 22.934 | 22.304 | 41.073 | 17.141 | 18.709 | 21.518 | **26.970** | **2.18** |
| **655** | 44.802 | 61.786 | 67.421 | 32.616 | 17.089 | 44.478 | 24.458 | 54.484 | 70.142 | 45.791 | 35.36 | 45.657 | 42.063 | 79.652 | 35.158 | 39.17 | 30.825 | **45.350** | **4.06** |
| **663** | 79.942 | 93.423 | 126.62 | 55.331 | 26.754 | 86.212 | 46.186 | 93.396 | 117.21 | 79.505 | 71.49 | 92.001 | 78.742 | 141.32 | 68.362 | 74.979 | 56.153 | **81.625** | **6.97** |
| **672** | 133.82 | 169.78 | 192.6 | 105.6 | 50.008 | 136.07 | 82.25 | 154.26 | 188.17 | 149.34 | 130.24 | 157.11 | 137.81 | 196.11 | 117.15 | 134.72 | 128.84 | **139.052** | **9.22** |
| **680** | 202.4 | 231.9 | 240.34 | 165.69 | 77.568 | 209.92 | 114.93 | 211.48 | 241.62 | 214.3 | 201.93 | 220.79 | 204.61 | 239.76 | 164.09 | 194.44 | 197.02 | **196.046** | **10.73** |
| **688** | 235.43 | 251.19 | 252.88 | 208.48 | 95.988 | 241.67 | 142.61 | 240.91 | 253.32 | 244.92 | 237.34 | 248.69 | 239.38 | 252.18 | 190.57 | 223.43 | 228.32 | **222.783** | **10.46** |
| **697** | 244.23 | 253.79 | 252.73 | 222.42 | 105.42 | 248.47 | 145.57 | 247.56 | 254.68 | 252.4 | 243.38 | 250.63 | 245.67 | 253.39 | 195.23 | 235.72 | 229.14 | **228.261** | **10.21** |
| **705** | 228.42 | 247.14 | 251.99 | 198.56 | 94.314 | 239.86 | 130.74 | 236.78 | 251.4 | 242.94 | 230.82 | 243.32 | 232.46 | 249.3 | 184.71 | 215.56 | 213.34 | **217.156** | **10.69** |
| **713** | 174.78 | 208.26 | 229.02 | 151.21 | 65.885 | 194.13 | 100.55 | 197.16 | 220.97 | 195.46 | 175.92 | 204.07 | 185.38 | 228.99 | 144.15 | 172.2 | 164 | **177.184** | **10.55** |
| **721** | 96.405 | 122.91 | 142.91 | 87.466 | 31.155 | 118.03 | 58.735 | 120.68 | 145.21 | 114.14 | 92.088 | 118.73 | 103.47 | 161.99 | 84.681 | 109.45 | 83.653 | **105.394** | **7.75** |
| **730** | 40.579 | 52.818 | 75.031 | 39.76 | 12.71 | 50.337 | 25.499 | 56.86 | 72.229 | 49.753 | 37.422 | 55.248 | 39.372 | 89.935 | 44.726 | 62.418 | 35.638 | **49.431** | **4.55** |
| **738** | 16.587 | 18.129 | 20.503 | 12.506 | 3.963 | 18.12 | 7.05 | 18.352 | 21.937 | 17.03 | 11.597 | 21.14 | 15.345 | 29.604 | 12.757 | 18.877 | 9.348 | **16.050** | **1.49** |

| **Leaf spongiform parenchyma cells** | | | | | | | | | | | | | | | | | | | | | | | | | | | | |
| --- | --- | --- | --- | --- | --- | --- | --- | --- | --- | --- | --- | --- | --- | --- | --- | --- | --- | --- | --- | --- | --- | --- | --- | --- | --- | --- | --- | --- |
| **Wavelength nm** |  | | **Intensity of emitted fluorescence** | | | | | | | | | | | | | | | | | | | | | | | | | |
|  | **1** | **2** | | **3** | **4** | **5** | **6** | **7** | **8** | **9** | **10** | **11** | **12** | **13** | **14** | **15** | **16** | **17** | **18** | **19** | **20** | **21** | **22** | **23** | **24** | **25** | **Average** | **SE** |
| **415** | 0.199 | 0.419 | | 0.144 | 0.173 | 0.551 | 0.207 | 0.334 | 0.317 | 0.124 | 0.448 | 0.123 | 0.338 | 0.508 | 0.308 | 0.168 | 0.81 | 0.567 | 0.003 | 0.03 | 0 | 0.059 | 0.005 | 0 | 0.013 | 0.072 | **0.028** | **0.009** |
| **423** | 4.113 | 1.828 | | 0.54 | 1.309 | 1.2 | 1.826 | 1.149 | 2.146 | 1.62 | 1.876 | 1.099 | 0.701 | 1.172 | 1.096 | 0.579 | 0.457 | 0.304 | 0.138 | 0.178 | 0.426 | 0.144 | 0.223 | 0.18 | 0.048 | 0.145 | **0.370** | **0.159** |
| **431** | 19.733 | 12.241 | | 11.899 | 8.48 | 6.498 | 9.588 | 2.604 | 17.797 | 13.977 | 14.569 | 11.555 | 4.95 | 11.489 | 8.08 | 3.597 | 4.3 | 4.567 | 3.113 | 2.89 | 4.075 | 3.326 | 3.357 | 3.475 | 2.02 | 3.416 | **3.870** | **0.401** |
| **440** | 31.916 | 20.22 | | 13.786 | 9.677 | 11.317 | 14.762 | 6.282 | 24.998 | 19.622 | 27.226 | 25.599 | 6.144 | 14.673 | 11.639 | 6.506 | 4.824 | 11.953 | 5.943 | 5.215 | 7.383 | 4.661 | 5.245 | 6.997 | 4.11 | 8.52 | **8.782** | **1.610** |
| **448** | 109.9 | 76.451 | | 56.238 | 37.957 | 47.103 | 53.025 | 20.94 | 86.149 | 69.459 | 86.832 | 77.04 | 23.92 | 47.521 | 37.609 | 22.306 | 19.831 | 35.252 | 32.556 | 33.306 | 44.056 | 27.44 | 29.374 | 36.151 | 23.41 | 32.02 | **39.611** | **3.421** |
| **456** | 184.47 | 138.72 | | 110.72 | 70.208 | 91.111 | 108.05 | 39.115 | 159.42 | 128.74 | 149.37 | 149.34 | 50.665 | 87.016 | 73.377 | 41.777 | 39.979 | 68.523 | 67.776 | 65.799 | 88.758 | 66.173 | 67.547 | 74.412 | 55.824 | 64.281 | **79.710** | **4.639** |
| **464** | 195.07 | 151.14 | | 125.86 | 82.904 | 113.89 | 120.94 | 49.387 | 162.84 | 141.49 | 162.73 | 151.36 | 56.643 | 93.497 | 79.597 | 50.046 | 49.337 | 79.731 | 77.109 | 68.848 | 89.99 | 66.792 | 72.759 | 79.539 | 56.517 | 68.364 | **86.424** | **4.923** |
| **472** | 194.14 | 147.75 | | 126.06 | 87.049 | 122.61 | 122.07 | 51.059 | 160.96 | 139.37 | 165.86 | 153.93 | 62.895 | 91.063 | 89.473 | 52.035 | 46.36 | 88.541 | 73.446 | 64.037 | 89.194 | 64.911 | 67.078 | 74.663 | 58.168 | 62.312 | **85.904** | **4.949** |
| **481** | 189.96 | 151.29 | | 124.58 | 92.976 | 114.56 | 114.55 | 47.609 | 156.81 | 131.16 | 160.7 | 155.81 | 61.783 | 85.252 | 86.496 | 52.37 | 41.418 | 87.46 | 67.879 | 57.988 | 76.786 | 56.953 | 67.67 | 67.726 | 48.686 | 63.333 | **77.700** | **4.333** |
| **489** | 179.48 | 134.78 | | 120.18 | 83.559 | 109.03 | 100.56 | 44.944 | 154.6 | 130.36 | 154.38 | 138.19 | 60.014 | 84.285 | 84.151 | 49.645 | 44.133 | 80.382 | 57.946 | 53.541 | 69.369 | 49.016 | 56.608 | 57.902 | 42.248 | 48.229 | **66.735** | **3.730** |
| **497** | 157.31 | 125.74 | | 93.069 | 65.446 | 87.866 | 88.961 | 28.569 | 128.63 | 113.67 | 122.06 | 116.66 | 46.593 | 59.055 | 62.462 | 40.058 | 39.009 | 61.854 | 42.177 | 38.284 | 46.312 | 36.601 | 41.558 | 41.892 | 27.4 | 36.895 | **47.403** | **2.605** |
| **505** | 130.45 | 100.81 | | 72.496 | 43.911 | 59.805 | 74.644 | 21.883 | 106.49 | 100.66 | 107.34 | 85.952 | 34.589 | 48.374 | 42.379 | 25.356 | 28.226 | 44.054 | 28.308 | 26.162 | 29.924 | 23.563 | 30.877 | 25.992 | 21.789 | 22.677 | **31.582** | **1.610** |
| **513** | 139.06 | 111.01 | | 68.037 | 44.179 | 55.139 | 81.815 | 21.192 | 113.7 | 106.6 | 106.04 | 90.023 | 33.793 | 46.975 | 45.308 | 25.192 | 31.122 | 48.557 | 27.265 | 26.159 | 33.981 | 25.508 | 30.245 | 27.758 | 20.32 | 23.77 | **30.791** | **1.526** |
| **522** | 156.57 | 126.17 | | 95.465 | 50.625 | 73.91 | 103.26 | 24.469 | 134.19 | 122.61 | 129.83 | 109.54 | 39.759 | 62.821 | 54.429 | 28.146 | 28.974 | 51.218 | 32.092 | 30.598 | 38.72 | 30.059 | 41.759 | 31.721 | 25.206 | 25.291 | **36.570** | **1.885** |
| **530** | 168.99 | 141.71 | | 95.949 | 59.412 | 80.835 | 114.32 | 26.482 | 139.38 | 138.61 | 136.43 | 116.15 | 42.261 | 58.636 | 67.236 | 34.118 | 35.302 | 59.486 | 31.425 | 31.787 | 37.483 | 33.953 | 37.089 | 30.924 | 26.772 | 30.229 | **37.566** | **2.004** |
| **538** | 151.89 | 126.53 | | 92.371 | 51.955 | 61.459 | 92.299 | 22.958 | 130.88 | 124.24 | 117.37 | 98.582 | 40.2 | 53.371 | 58.717 | 29.401 | 28.538 | 53.653 | 27.234 | 26.026 | 30.502 | 28.364 | 31.966 | 25.819 | 20.889 | 23.52 | **31.362** | **1.800** |
| **546** | 137.83 | 107.11 | | 79.54 | 50.277 | 61.587 | 81.565 | 22.283 | 114.3 | 120.4 | 103.63 | 84.805 | 31.785 | 49.508 | 47.949 | 25.549 | 30.785 | 53.239 | 20.39 | 22.106 | 23.81 | 21.614 | 28.385 | 20.16 | 16.337 | 15.916 | **25.259** | **1.627** |
| **554** | 120.65 | 94.286 | | 66.993 | 36.13 | 52.573 | 72.452 | 16.994 | 95.089 | 97.124 | 96.184 | 71.075 | 30.952 | 39.361 | 43.588 | 22.432 | 25.586 | 41.679 | 15.812 | 17.488 | 17.255 | 15.716 | 21.67 | 14.836 | 12.217 | 16.343 | **19.871** | **1.363** |
| **563** | 103.54 | 87.031 | | 64.547 | 34.365 | 45.902 | 69.567 | 18.929 | 89.878 | 92.245 | 87.028 | 62.257 | 24.119 | 35.304 | 43.098 | 22.81 | 26.623 | 38.283 | 12.244 | 13.193 | 17.094 | 14.703 | 17.558 | 12.631 | 11 | 11.854 | **17.001** | **1.254** |
| **571** | 100.07 | 75.856 | | 46.924 | 31.214 | 44.114 | 57.501 | 13.181 | 72.844 | 77.944 | 67.426 | 57.766 | 23.009 | 28.565 | 35.016 | 20.507 | 21.799 | 38.21 | 9.631 | 8.984 | 11.895 | 10.635 | 12.899 | 8.78 | 7.365 | 7.125 | **12.982** | **1.110** |
| **579** | 77.856 | 64.916 | | 42.484 | 24.581 | 39.192 | 40.227 | 12.256 | 62.695 | 68.634 | 59.026 | 42.542 | 19.519 | 27.09 | 29.65 | 15.302 | 13.327 | 32.658 | 7.624 | 7.56 | 7.398 | 9.758 | 10.787 | 7.441 | 5.944 | 6.208 | **10.311** | **0.886** |
| **587** | 66.203 | 50.646 | | 41.188 | 23.418 | 32.164 | 38.976 | 9.349 | 51.462 | 58.418 | 43.593 | 35.825 | 14.553 | 18.896 | 26.393 | 15.708 | 12.787 | 27.817 | 5.379 | 6.094 | 6.838 | 7.457 | 8.111 | 5.209 | 4.396 | 7.104 | **8.192** | **0.744** |
| **596** | 56.045 | 45.59 | | 35.572 | 22.768 | 27.334 | 32.598 | 9.206 | 44.213 | 49.052 | 37.138 | 30.125 | 11.781 | 17.896 | 20.422 | 11.226 | 12.667 | 25.286 | 3.893 | 4.628 | 4.587 | 5.182 | 6.167 | 4.092 | 3.537 | 4.135 | **6.204** | **0.607** |
| **604** | 51.386 | 36.475 | | 25.748 | 17.154 | 18.309 | 30.268 | 7.792 | 39.956 | 46.914 | 31.553 | 24.257 | 10.257 | 14.076 | 19.032 | 11.618 | 12.009 | 22.645 | 2.868 | 3.204 | 3.246 | 4.322 | 4.754 | 3.073 | 2.803 | 4.416 | **4.600** | **0.467** |
| **612** | 44.652 | 30.244 | | 22.572 | 13.023 | 19.451 | 22.117 | 7.824 | 28.98 | 32.818 | 28.189 | 22.727 | 8.591 | 12.667 | 16.77 | 10.279 | 7.628 | 19.882 | 2.212 | 2.174 | 3.744 | 2.936 | 3.648 | 3.048 | 1.503 | 2.229 | **3.599** | **0.335** |
| **620** | 36.476 | 28.055 | | 20.584 | 10.151 | 18.596 | 19.01 | 6.68 | 25.793 | 27.981 | 23.048 | 18.15 | 9.246 | 12.009 | 14.333 | 9.629 | 8.974 | 17.031 | 1.588 | 2.965 | 2.526 | 2.559 | 3 | 2.219 | 1.648 | 3.302 | **2.876** | **0.253** |
| **628** | 31.974 | 28.709 | | 17.679 | 12.2 | 11.203 | 19.368 | 6.606 | 24.677 | 24.779 | 18.657 | 17.742 | 10.114 | 12.17 | 13.356 | 10.874 | 10.258 | 15.593 | 1.283 | 1.575 | 2.104 | 2.385 | 2.374 | 1.504 | 0.91 | 1.968 | **2.329** | **0.227** |
| **637** | 31.912 | 22.839 | | 18.911 | 12.976 | 10.345 | 19.583 | 6.867 | 22.961 | 24.56 | 23.259 | 21.049 | 11.966 | 12.975 | 18.859 | 11.765 | 11.946 | 15.205 | 1.28 | 1.102 | 1.459 | 1.063 | 1.77 | 1.18 | 1.186 | 2.25 | **1.947** | **0.181** |
| **645** | 35.963 | 38.503 | | 35.213 | 19.972 | 15.966 | 32.375 | 12.33 | 31.969 | 38.588 | 27.931 | 26.001 | 22.934 | 22.304 | 41.073 | 17.141 | 18.709 | 21.518 | 1.503 | 1.772 | 1.729 | 2.025 | 2.502 | 1.743 | 1.441 | 2.468 | **2.448** | **0.234** |
| **653** | 44.802 | 61.786 | | 67.421 | 32.616 | 17.089 | 44.478 | 24.458 | 54.484 | 70.142 | 45.791 | 35.36 | 45.657 | 42.063 | 79.652 | 35.158 | 39.17 | 30.825 | 2.202 | 3.723 | 2.71 | 3.3 | 3.072 | 3.151 | 2.948 | 2.427 | **4.129** | **0.509** |
| **661** | 79.942 | 93.423 | | 126.62 | 55.331 | 26.754 | 86.212 | 46.186 | 93.396 | 117.21 | 79.505 | 71.49 | 92.001 | 78.742 | 141.32 | 68.362 | 74.979 | 56.153 | 4.581 | 5.696 | 5.734 | 7.597 | 4.413 | 7.129 | 6.844 | 6.343 | **8.853** | **1.244** |
| **669** | 133.82 | 169.78 | | 192.6 | 105.6 | 50.008 | 136.07 | 82.25 | 154.26 | 188.17 | 149.34 | 130.24 | 157.11 | 137.81 | 196.11 | 117.15 | 134.72 | 128.84 | 11.354 | 15.056 | 14.071 | 17.004 | 11.396 | 14.536 | 19.813 | 15.041 | **21.511** | **3.163** |
| **678** | 202.4 | 231.9 | | 240.34 | 165.69 | 77.568 | 209.92 | 114.93 | 211.48 | 241.62 | 214.3 | 201.93 | 220.79 | 204.61 | 239.76 | 164.09 | 194.44 | 197.02 | 30.617 | 41.359 | 35.72 | 42.745 | 30.776 | 36.768 | 49.803 | 35.072 | **48.297** | **6.746** |
| **686** | 235.43 | 251.19 | | 252.88 | 208.48 | 95.988 | 241.67 | 142.61 | 240.91 | 253.32 | 244.92 | 237.34 | 248.69 | 239.38 | 252.18 | 190.57 | 223.43 | 228.32 | 58.751 | 76.867 | 62.109 | 68.432 | 52.156 | 61.902 | 80.562 | 63.083 | **79.104** | **9.267** |
| **694** | 244.23 | 253.79 | | 252.73 | 222.42 | 105.42 | 248.47 | 145.57 | 247.56 | 254.68 | 252.4 | 243.38 | 250.63 | 245.67 | 253.39 | 195.23 | 235.72 | 229.14 | 77.216 | 92.306 | 75.18 | 83.936 | 60.564 | 73.865 | 104.24 | 72.885 | **92.027** | **9.769** |
| **702** | 228.42 | 247.14 | | 251.99 | 198.56 | 94.314 | 239.86 | 130.74 | 236.78 | 251.4 | 242.94 | 230.82 | 243.32 | 232.46 | 249.3 | 184.71 | 215.56 | 213.34 | 51.4 | 62.159 | 51.857 | 60.377 | 47.48 | 55.968 | 76.741 | 57.26 | **70.206** | **8.959** |
| **710** | 174.78 | 208.26 | | 229.02 | 151.21 | 65.885 | 194.13 | 100.55 | 197.16 | 220.97 | 195.46 | 175.92 | 204.07 | 185.38 | 228.99 | 144.15 | 172.2 | 164 | 25.985 | 29.068 | 27.995 | 33.588 | 17.296 | 27.329 | 38.827 | 30.302 | **36.823** | **5.710** |
| **719** | 96.405 | 122.91 | | 142.91 | 87.466 | 31.155 | 118.03 | 58.735 | 120.68 | 145.21 | 114.14 | 92.088 | 118.73 | 103.47 | 161.99 | 84.681 | 109.45 | 83.653 | 7.985 | 9.689 | 8.962 | 9.644 | 6.603 | 9.302 | 13.006 | 10.781 | **12.446** | **2.150** |
| **727** | 40.579 | 52.818 | | 75.031 | 39.76 | 12.71 | 50.337 | 25.499 | 56.86 | 72.229 | 49.753 | 37.422 | 55.248 | 39.372 | 89.935 | 44.726 | 62.418 | 35.638 | 2.095 | 2.299 | 1.786 | 2.966 | 1.374 | 2.804 | 3.824 | 2.989 | **3.488** | **0.626** |
| **735** | 16.587 | 18.129 | | 20.503 | 12.506 | 3.963 | 18.12 | 7.05 | 18.352 | 21.937 | 17.03 | 11.597 | 21.14 | 15.345 | 29.604 | 12.757 | 18.877 | 9.348 | 0.542 | 0.253 | 0.317 | 0.394 | 0.078 | 0.69 | 0.572 | 0.312 | **0.618** | **0.111** |

| **Leaf glandular structure** | | | | | | | | | | | | | | | | | |  |
| --- | --- | --- | --- | --- | --- | --- | --- | --- | --- | --- | --- | --- | --- | --- | --- | --- | --- | --- |
|  | **Intensity of emitted fluorescence** | | | | | | | | | | | | | | | | | |
| **Wavelength nm** | **1** | | **2** | | **3** | **4** | | | **5** | **6** | **7** | **8** | **9** | **10** | **11** | **Average** | **SE** | |
| **415** | **0.003** | | 0.03 | | 0.004 | 0.012 | | | 0.001 | 0 | 0.016 | 0 | 0.029 | 0 | 0.003 | **0.009** | **0.00** | |
| **423** | **0.215** | | 0.225 | | 0.103 | 0.514 | | | 0.138 | 0.123 | 0.259 | 0.117 | 0.107 | 0.145 | 0.096 | **0.186** | **0.04** | |
| **431** | 5.767 | | 6.545 | | 4.594 | 13.247 | | | 3.898 | 2.559 | 4.413 | 2.274 | 1.799 | 3.242 | 1.957 | **4.572** | **0.98** | |
| **440** | 15.148 | | 16.325 | | 12.309 | 29.501 | | | 10.545 | 8.279 | 9.047 | 4.193 | 3.49 | 5.833 | 5.225 | **10.900** | **2.26** | |
| **448** | 80.465 | | 85.382 | | 63.921 | 121.97 | | | 56.91 | 45.388 | 52.592 | 32.431 | 31.363 | 38.389 | 37.93 | **58.795** | **8.37** | |
| **456** | 164.53 | | 173.21 | | 131.12 | 208.14 | | | 118.21 | 95.097 | 113.52 | 76.81 | 75.982 | 88.241 | 80.245 | **120.464** | **13.40** | |
| **464** | 186.87 | | 199.42 | | 146.42 | 212.59 | | | 132.01 | 108.34 | 132.27 | 94.354 | 92.342 | 105.52 | 90.528 | **136.424** | **13.49** | |
| **472** | 191.16 | | 205.75 | | 146.9 | 213.23 | | | 135.72 | 111.48 | 145.61 | 109 | 102.54 | 117.95 | 90.125 | **142.679** | **12.96** | |
| **481** | 181.46 | | 192.43 | | 133.08 | 202.06 | | | 122.47 | 99.373 | 146.81 | 109.59 | 105.72 | 119.87 | 84.661 | **136.139** | **11.98** | |
| **489** | 155.16 | | 162.33 | | 109.14 | 176.86 | | | 103.92 | 81.755 | 136.08 | 106.15 | 101.55 | 113.07 | 68.025 | **119.458** | **10.29** | |
| **497** | 106.65 | | 113.21 | | 72.633 | 119.17 | | | 67.667 | 54.619 | 105.9 | 79.708 | 74.657 | 84.289 | 44.766 | **83.934** | **7.38** | |
| **505** | 65.13 | | 69.214 | | 42.567 | 68.72 | | | 39.804 | 30.218 | 72.442 | 54.044 | 50.137 | 59.128 | 25.224 | **52.421** | **4.91** | |
| **513** | 56.614 | | 59.592 | | 36.127 | 63.098 | | | 37.546 | 27.488 | 73.623 | 55.249 | 52.434 | 58.188 | 21.963 | **49.266** | **4.86** | |
| **522** | 63.813 | | 67.243 | | 40.126 | 67.321 | | | 39.512 | 28.563 | 88.819 | 66.851 | 63.298 | 72.855 | 24.527 | **56.630** | **6.11** | |
| **530** | 60.392 | | 64.824 | | 39.3 | 62.972 | | | 40.847 | 28.14 | 94.598 | 73.127 | 69.286 | 77.242 | 23.506 | **57.658** | **6.65** | |
| **538** | 46.713 | | 51.93 | | 31.644 | 48.282 | | | 31.435 | 21.087 | 80.336 | 64.427 | 60.622 | 68.638 | 17.846 | **47.542** | **6.10** | |
| **546** | 38.729 | | 43.477 | | 23.985 | 38.381 | | | 25.12 | 16.156 | 73.106 | 57.505 | 52.026 | 59.528 | 13.992 | **40.182** | **5.77** | |
| **554** | 28.897 | | 32.701 | | 17.074 | 29.038 | | | 19.413 | 12.565 | 63.748 | 49.516 | 46.534 | 52.665 | 10.494 | **32.968** | **5.38** | |
| **563** | 25.679 | | 27.363 | | 14.497 | 24.344 | | | 15.875 | 9.878 | 58.091 | 44.274 | 42.147 | 47.709 | 8.486 | **28.940** | **5.05** | |
| **571** | 18.695 | | 21.469 | | 10.26 | 17.894 | | | 12.555 | 6.968 | 48.338 | 37.082 | 35.571 | 40.399 | 6.943 | **23.289** | **4.40** | |
| **579** | 13.749 | | 15.146 | | 6.963 | 13.074 | | | 8.303 | 4.605 | 37.731 | 31.11 | 28.325 | 33.119 | 4.836 | **17.906** | **3.71** | |
| **587** | 9.645 | | 11.496 | | 5.21 | 9.522 | | | 6.547 | 3.204 | 34.249 | 25.067 | 22.987 | 27.294 | 3.292 | **14.410** | **3.30** | |
| **596** | 7.663 | | 8.123 | | 3.786 | 7.162 | | | 5.015 | 2.607 | 27.405 | 21.042 | 18.444 | 22.627 | 2.734 | **11.510** | **2.73** | |
| **604** | 5.077 | | 5.57 | | 2.704 | 4.157 | | | 3.317 | 1.777 | 20.943 | 16.519 | 14.08 | 18.256 | 1.586 | **8.544** | **2.21** | |
| **612** | 3.826 | | 3.868 | | 1.608 | 3.238 | | | 2.523 | 1.269 | 17.395 | 12.31 | 9.592 | 12.64 | 1.004 | **6.298** | **1.71** | |
| **620** | 2.396 | | 2.548 | | 1.539 | 2.412 | | | 1.803 | 1.075 | 13.854 | 10.158 | 8.546 | 9.943 | 0.784 | **5.005** | **1.40** | |
| **628** | 1.851 | | 1.941 | | 0.9 | 1.638 | | | 1.133 | 0.716 | 10.018 | 6.992 | 6.282 | 7.765 | 0.506 | **3.613** | **1.04** | |
| **637** | 1.172 | | 1.646 | | 0.834 | 1.227 | | | 0.888 | 0.305 | 7.742 | 4.84 | 4.377 | 6.005 | 0.433 | **2.679** | **0.78** | |
| **645** | 1.103 | | 0.969 | | 0.596 | 0.76 | | | 0.828 | 0.297 | 6.6 | 4.033 | 3.826 | 4.555 | 0.28 | **2.168** | **0.66** | |
| **653** | 0.784 | | 0.529 | | 0.643 | 0.842 | | | 0.984 | 0.438 | 3.656 | 3.088 | 2.077 | 3.093 | 0.317 | **1.496** | **0.37** | |
| **661** | 0.621 | | 0.349 | | 1.273 | 0.962 | | | 2.011 | 0.39 | 3.187 | 1.88 | 1.242 | 2.237 | 0.573 | **1.339** | **0.27** | |
| **669** | 1.388 | | 0.415 | | 2.921 | 1.618 | | | 5.573 | 0.876 | 2.106 | 1.418 | 1.073 | 1.407 | 1.332 | **1.830** | **0.42** | |
| **678** | 3.263 | | 0.434 | | 9.387 | 3.628 | | | 14.114 | 3.315 | 1.509 | 0.957 | 0.896 | 1.661 | 3.534 | **3.882** | **1.26** | |
| **686** | 6.937 | | 0.507 | | 18.731 | 6.195 | | | 26.124 | 5.567 | 0.968 | 0.767 | 0.653 | 1.391 | 6.764 | **6.782** | **2.51** | |
| **694** | 8.2 | | 0.428 | | 21.745 | 8.07 | | | 29.429 | 6.313 | 0.846 | 0.347 | 0.527 | 1.028 | 7.391 | **7.666** | **2.89** | |
| **702** | 4.365 | | 0.292 | | 12.753 | 4.694 | | | 18.488 | 3.702 | 0.282 | 0.224 | 0.253 | 0.441 | 4.176 | **4.515** | **1.79** | |
| **710** | 1.765 | | 0.077 | | 4.129 | 1.365 | | | 6.121 | 1.422 | 0.172 | 0.124 | 0.154 | 0.148 | 1.258 | **1.521** | **0.59** | |
| **719** | 0.338 | | 0.041 | | 0.917 | 0.354 | | | 1.469 | 0.339 | 0.024 | 0.056 | 0.084 | 0.081 | 0.232 | **0.358** | **0.14** | |
| **727** | 0.105 | | 0.007 | | 0.181 | 0.09 | | | 0.298 | 0.109 | 0.018 | 0.005 | 0.021 | 0.017 | 0.113 | **0.088** | **0.03** | |
| **735** | 0 | | 0.007 | | 0.05 | 0 | | | 0.043 | 0.021 | 0 | 0.008 | 0.036 | 0 | 0.019 | **0.017** | **0.01** | |
| **Root cortical cells** | | | | | | | |  | | | | | | | | | | |
|  | | **Intensity of emitted fluorescence** | | | | |  | | | | | | | | | | | |
| **Wavelength nm** | | **Average** | | **SE** | | | |  | | | | | | | | | | |
| **415** | | 0.009 | | 0.00 | | | |  | | | | | | | | | | |
| **423** | | 0.186 | | 0.04 | | | |  | | | | | | | | | | |
| **431** | | 4.572 | | 0.98 | | | |  | | | | | | | | | | |
| **440** | | 10.900 | | 2.26 | | | |  | | | | | | | | | | |
| **448** | | 58.795 | | 8.37 | | | |  | | | | | | | | | | |
| **456** | | 120.464 | | 13.40 | | | |  | | | | | | | | | | |
| **464** | | 136.424 | | 13.49 | | | |  | | | | | | | | | | |
| **472** | | 142.679 | | 12.96 | | | |  | | | | | | | | | | |
| **481** | | 136.139 | | 11.98 | | | |  | | | | | | | | | | |
| **489** | | 119.458 | | 10.29 | | | |  | | | | | | | | | | |
| **497** | | 83.934 | | 7.38 | | | |  | | | | | | | | | | |
| **505** | | 52.421 | | 4.91 | | | |  | | | | | | | | | | |
| **513** | | 49.266 | | 4.86 | | | |  | | | | | | | | | | |
| **522** | | 56.630 | | 6.11 | | | |  | | | | | | | | | | |
| **530** | | 57.658 | | 6.65 | | | |  | | | | | | | | | | |
| **538** | | 47.542 | | 6.10 | | | |  | | | | | | | | | | |
| **546** | | 40.182 | | 5.77 | | | |  | | | | | | | | | | |
| **554** | | 32.968 | | 5.38 | | | |  | | | | | | | | | | |
| **563** | | 28.940 | | 5.05 | | | |  | | | | | | | | | | |
| **571** | | 23.289 | | 4.40 | | | |  | | | | | | | | | | |
| **579** | | 17.906 | | 3.71 | | | |  | | | | | | | | | | |
| **587** | | 14.410 | | 3.30 | | | |  | | | | | | | | | | |
| **596** | | 11.510 | | 2.73 | | | |  | | | | | | | | | | |
| **604** | | 8.544 | | 2.21 | | | |  | | | | | | | | | | |
| **612** | | 6.298 | | 1.71 | | | |  | | | | | | | | | | |
| **620** | | 5.005 | | 1.40 | | | |  | | | | | | | | | | |
| **628** | | 3.613 | | 1.04 | | | |  | | | | | | | | | | |
| **637** | | 2.679 | | 0.78 | | | |  | | | | | | | | | | |
| **645** | | 2.168 | | 0.66 | | | |  | | | | | | | | | | |
| **653** | | 1.496 | | 0.37 | | | |  | | | | | | | | | | |
| **661** | | 1.339 | | 0.27 | | | |  | | | | | | | | | | |
| **669** | | 1.830 | | 0.42 | | | |  | | | | | | | | | | |
| **678** | | 3.882 | | 1.26 | | | |  | | | | | | | | | | |
| **686** | | 6.782 | | 2.51 | | | |  | | | | | | | | | | |
| **694** | | 7.666 | | 2.89 | | | |  | | | | | | | | | | |
| **702** | | 4.515 | | 1.79 | | | |  | | | | | | | | | | |
| **710** | | 1.521 | | 0.59 | | | |  | | | | | | | | | | |
| **719** | | 0.358 | | 0.14 | | | |  | | | | | | | | | | |
| **727** | | 0.088 | | 0.03 | | | |  | | | | | | | | | | |
| **735** | | 0.017 | | 0.01 | | | |  | | | | | | | | | | |

| **Flower extracts** | | | | | | | | | | | | |
| --- | --- | --- | --- | --- | --- | --- | --- | --- | --- | --- | --- | --- |
|  | **Intensity of emitted fluorescence** | | | | | | | | | | |  |
| **Wavelength nm** | **1** | **2** | **3** | **4** | **5** | **6** | **7** | **8** | **9** | **Average** | **SE** |  |
| **415** | 5.461 | 5.434 | 5.458 | 5.399 | 5.412 | 5.424 | 5.471 | 5.438 | 5.458 | **5.439** | **0.01** |  |
| **423** | 6.481 | 6.69 | 6.638 | 6.589 | 6.525 | 6.533 | 6.511 | 6.62 | 6.549 | **6.571** | **0.07** |  |
| **432** | 10.317 | 10.236 | 10.896 | 10.479 | 10.058 | 10.064 | 10.482 | 10.114 | 10.454 | **10.344** | **0.27** |  |
| **440** | 15.061 | 15.782 | 16.739 | 16.099 | 16.271 | 15.736 | 15.844 | 15.375 | 15.695 | **15.845** | **0.49** |  |
| **448** | 44.002 | 47.862 | 48.756 | 47.33 | 47.609 | 45.585 | 45.693 | 43.546 | 45.146 | **46.170** | **1.81** |  |
| **456** | 68.101 | 74.738 | 77.697 | 75.503 | 74.992 | 69.563 | 71.001 | 66.754 | 69.299 | **71.961** | **3.84** |  |
| **465** | 80.46 | 85.872 | 89.279 | 87.73 | 84.511 | 79.222 | 80.791 | 75.108 | 77.495 | **82.274** | **4.82** |  |
| **473** | 81.679 | 89.679 | 94.11 | 92.392 | 85.862 | 80.026 | 84.79 | 77.005 | 78.345 | **84.876** | **6.17** |  |
| **481** | 81.003 | 87.431 | 91.516 | 89.991 | 84.624 | 76.898 | 82.116 | 76.498 | 75.145 | **82.802** | **6.01** |  |
| **490** | 71.379 | 75.819 | 78.433 | 78.546 | 72.463 | 66.736 | 71.031 | 65.564 | 65.529 | **71.722** | **5.13** |  |
| **498** | 51.471 | 55.786 | 57.897 | 56.364 | 52.614 | 48.392 | 51.66 | 48.235 | 47.356 | **52.197** | **3.83** |  |
| **506** | 38.921 | 42.509 | 43.707 | 42.594 | 38.602 | 35.973 | 39.23 | 36.264 | 35.603 | **39.267** | **3.06** |  |
| **514** | 47.408 | 49.752 | 50.479 | 50.457 | 45.965 | 43.302 | 46.072 | 43.095 | 42.068 | **46.511** | **3.26** |  |
| **523** | 52.268 | 54.288 | 57.48 | 56.556 | 50.727 | 46.19 | 52.101 | 48.656 | 46.702 | **51.663** | **4.03** |  |
| **531** | 49.987 | 53.099 | 54.732 | 54.292 | 49.716 | 45.311 | 50.267 | 45.683 | 44.512 | **49.733** | **3.88** |  |
| **539** | 46.713 | 50.699 | 51.436 | 50.74 | 45.654 | 42.451 | 46.54 | 43.062 | 41.453 | **46.528** | **3.78** |  |
| **548** | 41.784 | 46.226 | 45.836 | 46.128 | 41.969 | 38.773 | 42.341 | 39.225 | 37.077 | **42.151** | **3.39** |  |
| **556** | 39.514 | 41.593 | 43.992 | 43.566 | 39.643 | 37.071 | 39.782 | 37.036 | 36.33 | **39.836** | **2.79** |  |
| **564** | 37.702 | 40.984 | 41.429 | 41.246 | 37.748 | 34.386 | 37.162 | 34.995 | 34.908 | **37.840** | **2.82** |  |
| **572** | 32.634 | 36.866 | 37.393 | 37.127 | 33.728 | 31.383 | 33.152 | 32.012 | 30.717 | **33.890** | **2.59** |  |
| **581** | 32.409 | 34.843 | 37.019 | 35.974 | 32.82 | 30.546 | 32.359 | 30.032 | 30.158 | **32.907** | **2.55** |  |
| **589** | 30.88 | 33.675 | 35.307 | 35.021 | 32.302 | 30.486 | 31.975 | 29.759 | 30.207 | **32.179** | **2.08** |  |
| **597** | 32.917 | 35.539 | 36.258 | 35.493 | 32.867 | 31.518 | 33.133 | 30.733 | 29.751 | **33.134** | **2.27** |  |
| **605** | 32.258 | 36.452 | 37.609 | 38.152 | 34.561 | 33.383 | 34.091 | 31.819 | 32.023 | **34.483** | **2.41** |  |
| **614** | 38.073 | 41.737 | 43.836 | 43.34 | 39.597 | 37.745 | 38.809 | 35.437 | 37.302 | **39.542** | **2.86** |  |
| **622** | 40.728 | 44.687 | 46.722 | 47.43 | 43.367 | 40.524 | 41.094 | 39.111 | 39.783 | **42.605** | **3.07** |  |
| **630** | 42.696 | 46.106 | 48.632 | 48.208 | 44.798 | 42.757 | 43.394 | 40 | 41.185 | **44.197** | **2.99** |  |
| **639** | 44.148 | 48.669 | 50.332 | 51.157 | 48.22 | 45.874 | 45.634 | 42.479 | 44.276 | **46.754** | **2.98** |  |
| **647** | 40.703 | 45.657 | 48.491 | 49.453 | 45.248 | 43.989 | 43.353 | 39.654 | 42.7 | **44.361** | **3.26** |  |
| **655** | 35.334 | 39.504 | 42.074 | 41.367 | 38.817 | 37.998 | 37.071 | 34.273 | 36.543 | **38.109** | **2.62** |  |
| **663** | 33.918 | 36.465 | 38.982 | 39.214 | 36.405 | 35.253 | 35.619 | 32.864 | 33.982 | **35.856** | **2.19** |  |
| **672** | 30.307 | 32.598 | 34.12 | 35.191 | 32.718 | 32.218 | 30.644 | 29.262 | 29.915 | **31.886** | **2.00** |  |
| **680** | 25.005 | 27.33 | 29.533 | 29.509 | 27.594 | 27.378 | 26.564 | 25.045 | 26.249 | **27.134** | **1.65** |  |
| **688** | 17.808 | 18.958 | 20.115 | 20.309 | 18.741 | 18.394 | 17.554 | 17.011 | 17.58 | **18.497** | **1.15** |  |
| **697** | 13.261 | 14.6 | 14.902 | 15.033 | 14.244 | 14.368 | 13.469 | 13.234 | 13.631 | **14.082** | **0.70** |  |
| **705** | 7.415 | 7.341 | 7.458 | 7.698 | 7.544 | 7.646 | 7.28 | 7.508 | 7.348 | **7.471** | **0.05** |  |
| **713** | 6.234 | 6.188 | 6.4 | 6.589 | 6.347 | 6.327 | 6.423 | 6.304 | 6.242 | **6.339** | **0.12** |  |
| **721** | 5.556 | 5.452 | 5.455 | 5.51 | 5.496 | 5.487 | 5.461 | 5.488 | 5.491 | **5.488** | **0.03** |  |
| **730** | 5.468 | 5.436 | 5.461 | 5.448 | 5.412 | 5.449 | 5.456 | 5.489 | 5.498 | **5.457** | **0.03** |  |
| **738** | 5.493 | 5.416 | 5.5 | 5.452 | 5.45 | 5.44 | 5.445 | 5.451 | 5.427 | **5.453** | **0.03** |  |

| **Leaf extracts** | | | | | | | | | |  |
| --- | --- | --- | --- | --- | --- | --- | --- | --- | --- | --- |
|  | **Intensity of emitted fluorescence** | | | | | | | | | |
| **Wavelength nm** | **1** | **2** | **3** | **4** | **5** | **6** | **7** | **Average** | **SE** | |
| **415** | 5.456 | 5.494 | 5.456 | 5.479 | 5.456 | 5.439 | 5.464 | **5.463** | **0.01** | |
| **423** | 6.605 | 6.605 | 6.605 | 6.402 | 6.475 | 6.526 | 6.437 | **6.522** | **0.03** | |
| **432** | 11.1 | 11.654 | 11.1 | 10.425 | 10.764 | 10.454 | 10.452 | **10.850** | **0.17** | |
| **440** | 17.397 | 18.517 | 17.397 | 16.156 | 17.073 | 16.589 | 16.36 | **17.070** | **0.30** | |
| **448** | 50.788 | 54.386 | 50.788 | 45.865 | 49.799 | 47.855 | 47.45 | **49.562** | **1.06** | |
| **456** | 95.07 | 101.84 | 95.07 | 83.293 | 94.526 | 87.857 | 87.399 | **92.151** | **2.37** | |
| **465** | 116.7 | 124.05 | 116.7 | 103.33 | 112 | 104.71 | 102.64 | **111.447** | **3.10** | |
| **473** | 128.24 | 136.24 | 128.24 | 112.12 | 123.1 | 113.62 | 112.33 | **121.984** | **3.60** | |
| **481** | 128.33 | 136.6 | 128.33 | 109.78 | 124.24 | 111.65 | 109.65 | **121.226** | **4.09** | |
| **490** | 116.93 | 126.3 | 116.93 | 100.17 | 114.07 | 102.92 | 101.17 | **111.213** | **3.76** | |
| **498** | 88.916 | 96.476 | 88.916 | 76.48 | 86.028 | 77.262 | 76.895 | **84.425** | **2.92** | |
| **506** | 63.565 | 68.865 | 63.565 | 56.22 | 61.83 | 55.954 | 55.826 | **60.832** | **1.89** | |
| **514** | 67.279 | 71.766 | 67.279 | 58.458 | 63.819 | 59.21 | 58.678 | **63.784** | **1.97** | |
| **523** | 75.199 | 82.865 | 75.199 | 67.14 | 71.62 | 67 | 66.874 | **72.271** | **2.25** | |
| **531** | 78.027 | 84.486 | 78.027 | 68.913 | 73.233 | 68.973 | 68.435 | **74.299** | **2.31** | |
| **539** | 71.799 | 76.824 | 71.799 | 62.176 | 66.69 | 62.889 | 62.951 | **67.875** | **2.15** | |
| **548** | 64.608 | 68.737 | 64.608 | 57.441 | 61.059 | 57.53 | 58.059 | **61.720** | **1.66** | |
| **556** | 58.185 | 63.075 | 58.185 | 52.586 | 54.617 | 52.634 | 53.023 | **56.044** | **1.49** | |
| **564** | 52.39 | 57.624 | 52.39 | 46.341 | 49.298 | 47.384 | 47.67 | **50.442** | **1.50** | |
| **572** | 47.47 | 51.714 | 47.47 | 41.266 | 45.025 | 43.129 | 43.261 | **45.619** | **1.34** | |
| **581** | 41.328 | 44.786 | 41.328 | 37.281 | 38.099 | 37.843 | 38.659 | **39.903** | **1.02** | |
| **589** | 37.654 | 40.301 | 37.654 | 33.118 | 34.592 | 34.58 | 34.248 | **36.021** | **0.97** | |
| **597** | 33.511 | 36.096 | 33.511 | 32.676 | 30.695 | 30.958 | 31.364 | **32.687** | **0.72** | |
| **605** | 28.991 | 31.93 | 28.991 | 27.996 | 26.416 | 27.812 | 27.184 | **28.474** | **0.67** | |
| **614** | 26.121 | 28.367 | 26.121 | 25.479 | 23.006 | 24.7 | 24.402 | **25.457** | **0.64** | |
| **622** | 23.797 | 25.358 | 23.797 | 21.641 | 20.866 | 22.482 | 22.003 | **22.849** | **0.58** | |
| **630** | 20.542 | 21.51 | 20.542 | 19.678 | 18.031 | 19.365 | 19.447 | **19.874** | **0.42** | |
| **639** | 18.8 | 19.616 | 18.8 | 18.544 | 16.696 | 17.34 | 17.91 | **18.244** | **0.38** | |
| **647** | 17.115 | 17.932 | 17.115 | 16.515 | 15.027 | 16.081 | 16.166 | **16.564** | **0.35** | |
| **655** | 15.547 | 16.467 | 15.547 | 14.431 | 13.577 | 14.433 | 14.336 | **14.905** | **0.37** | |
| **663** | 13.882 | 15.191 | 13.882 | 14.272 | 12.432 | 13.112 | 13.969 | **13.820** | **0.33** | |
| **672** | 14.018 | 16.051 | 14.018 | 15.005 | 12.042 | 13.482 | 14.636 | **14.179** | **0.48** | |
| **680** | 14.124 | 16.317 | 14.124 | 16.147 | 12.364 | 13.746 | 15.185 | **14.572** | **0.53** | |
| **688** | 11.967 | 14.279 | 11.967 | 15.104 | 11.39 | 12.485 | 13.853 | **13.006** | **0.53** | |
| **697** | 10.198 | 11.587 | 10.198 | 12.556 | 9.742 | 10.267 | 11.574 | **10.875** | **0.39** | |
| **705** | 7.549 | 8.119 | 7.549 | 8.129 | 7.423 | 7.618 | 8.079 | **7.781** | **0.12** | |
| **713** | 6.701 | 6.962 | 6.701 | 6.963 | 6.567 | 6.557 | 6.694 | **6.735** | **0.06** | |
| **721** | 5.727 | 5.82 | 5.727 | 5.86 | 5.709 | 5.74 | 5.746 | **5.761** | **0.02** | |
| **730** | 5.476 | 5.514 | 5.476 | 5.497 | 5.438 | 5.496 | 5.517 | **5.488** | **0.01** | |
| **738** | 5.426 | 5.462 | 5.425 | 5.507 | 5.499 | 5.423 | 5.419 | **5.452** | **0.01** | |

| **Leaf border extracts** | | | | | | | | | | | | | | | | | | | | | | |
| --- | --- | --- | --- | --- | --- | --- | --- | --- | --- | --- | --- | --- | --- | --- | --- | --- | --- | --- | --- | --- | --- | --- |
|  |  | | **Intensity of emitted fluorescence** | | | | | | | | | | | | | | | | | | |  |
| **Wavelength nm** | **1** | **2** | | **3** | **4** | **5** | **6** | **7** | **8** | **9** | **10** | **11** | **12** | **13** | **14** | **15** | **16** | **17** | **18** | **Average** | **SE** | |
| **415** | 6.154 | 6.315 | | 6.074 | 6.199 | 6.193 | 6.134 | 6.134 | 6.117 | 6.249 | 5.958 | 6.007 | 6.266 | 5.886 | 6.260 | 6.020 | 6.074 | 6.198 | 6.210 | **6.136** | **0.115** | |
| **423** | 8.288 | 7.874 | | 8.807 | 8.770 | 8.924 | 9.005 | 9.005 | 9.126 | 8.775 | 8.540 | 7.609 | 9.037 | 9.498 | 7.956 | 8.802 | 8.914 | 9.078 | 8.064 | **8.671** | **0.509** | |
| **431** | 25.674 | 32.300 | | 30.843 | 34.436 | 29.698 | 33.449 | 33.449 | 32.776 | 35.805 | 26.183 | 28.291 | 27.842 | 27.194 | 28.152 | 29.776 | 30.850 | 33.006 | 31.592 | **30.629** | **2.954** | |
| **440** | 42.014 | 48.163 | | 49.933 | 52.446 | 47.089 | 50.769 | 50.769 | 52.271 | 54.380 | 40.719 | 42.449 | 43.205 | 42.844 | 41.500 | 46.877 | 47.701 | 51.203 | 47.636 | **47.331** | **4.297** | |
| **448** | 94.944 | 106.688 | | 106.528 | 113.976 | 104.856 | 110.928 | 110.928 | 110.968 | 116.640 | 93.600 | 90.784 | 100.528 | 97.296 | 95.584 | 105.440 | 107.264 | 111.184 | 114.264 | **105.133** | **7.887** | |
| **456** | 152.888 | 163.912 | | 166.928 | 171.216 | 163.648 | 169.992 | 169.992 | 167.560 | 172.688 | 148.496 | 147.456 | 160.088 | 155.600 | 149.912 | 163.992 | 163.312 | 164.984 | 170.768 | **162.413** | **8.205** | |
| **464** | 159.384 | 170.768 | | 171.912 | 175.552 | 168.264 | 174.872 | 174.872 | 173.176 | 177.728 | 154.552 | 156.056 | 164.272 | 161.496 | 157.488 | 170.976 | 173.120 | 171.224 | 176.624 | **168.463** | **7.578** | |
| **472** | 159.936 | 172.104 | | 171.840 | 176.256 | 168.680 | 175.496 | 175.496 | 171.928 | 177.072 | 157.496 | 155.856 | 166.640 | 160.832 | 158.656 | 170.872 | 171.376 | 172.384 | 176.928 | **168.880** | **7.188** | |
| **481** | 152.080 | 164.464 | | 169.232 | 171.336 | 163.368 | 169.808 | 169.808 | 168.032 | 173.536 | 151.968 | 151.464 | 160.904 | 157.392 | 153.128 | 163.200 | 165.904 | 169.344 | 171.984 | **163.720** | **7.542** | |
| **489** | 148.728 | 160.232 | | 161.624 | 164.928 | 156.880 | 163.264 | 163.264 | 162.936 | 166.440 | 143.120 | 142.128 | 158.576 | 151.920 | 144.824 | 158.128 | 158.736 | 163.248 | 165.224 | **157.456** | **7.910** | |
| **497** | 129.432 | 144.168 | | 145.496 | 149.552 | 141.992 | 147.920 | 147.920 | 144.984 | 152.568 | 126.408 | 126.064 | 137.968 | 134.784 | 130.344 | 142.528 | 143.376 | 145.264 | 144.936 | **140.872** | **8.126** | |
| **505** | 98.752 | 109.328 | | 112.312 | 116.216 | 108.792 | 115.792 | 115.792 | 113.144 | 117.816 | 96.720 | 94.000 | 106.200 | 102.632 | 96.936 | 104.576 | 110.544 | 112.624 | 113.088 | **108.070** | **7.520** | |
| **513** | 76.614 | 89.760 | | 89.696 | 94.848 | 86.920 | 91.760 | 91.760 | 93.264 | 98.528 | 75.366 | 74.203 | 86.744 | 80.496 | 78.218 | 88.032 | 88.624 | 91.136 | 93.544 | **87.195** | **7.208** | |
| **522** | 85.256 | 96.312 | | 99.768 | 102.792 | 97.056 | 102.696 | 102.696 | 101.608 | 105.224 | 83.080 | 82.064 | 95.608 | 90.424 | 87.480 | 97.256 | 96.264 | 101.040 | 101.704 | **96.018** | **7.307** | |
| **530** | 89.288 | 101.360 | | 102.464 | 106.296 | 98.784 | 108.984 | 108.984 | 106.464 | 109.440 | 90.232 | 88.744 | 100.384 | 98.136 | 89.056 | 100.928 | 103.024 | 106.288 | 102.424 | **100.627** | **7.071** | |
| **538** | 86.784 | 99.440 | | 102.888 | 105.344 | 97.880 | 105.472 | 105.472 | 103.632 | 107.016 | 85.392 | 79.019 | 97.448 | 90.904 | 86.976 | 97.040 | 98.840 | 103.744 | 102.728 | **97.557** | **8.315** | |
| **546** | 77.623 | 88.336 | | 88.512 | 94.368 | 88.672 | 94.600 | 94.600 | 92.344 | 96.416 | 77.828 | 74.477 | 84.864 | 79.528 | 79.908 | 89.144 | 87.464 | 90.704 | 96.920 | **87.573** | **7.048** | |
| **554** | 68.400 | 79.794 | | 82.912 | 86.544 | 78.389 | 84.888 | 84.888 | 85.104 | 88.168 | 67.754 | 68.594 | 77.450 | 72.422 | 71.853 | 82.376 | 85.160 | 82.688 | 85.128 | **79.584** | **6.869** | |
| **563** | 65.949 | 75.296 | | 75.002 | 79.362 | 73.035 | 78.161 | 78.161 | 78.494 | 80.960 | 61.913 | 62.916 | 72.515 | 67.617 | 65.485 | 71.234 | 72.962 | 77.902 | 78.767 | **73.096** | **6.024** | |
| **571** | 60.872 | 68.750 | | 71.071 | 71.980 | 67.599 | 70.773 | 70.773 | 72.111 | 76.054 | 57.750 | 59.610 | 65.486 | 62.675 | 60.078 | 67.409 | 69.694 | 68.909 | 71.750 | **67.408** | **5.194** | |
| **579** | 50.510 | 59.051 | | 61.909 | 65.022 | 61.105 | 63.861 | 63.861 | 64.234 | 66.685 | 48.618 | 49.630 | 56.445 | 58.350 | 49.198 | 54.678 | 59.958 | 63.469 | 61.069 | **58.758** | **5.923** | |
| **587** | 42.418 | 51.694 | | 53.224 | 56.462 | 49.734 | 53.911 | 53.911 | 55.459 | 57.732 | 42.618 | 44.428 | 49.402 | 49.206 | 45.146 | 48.767 | 52.113 | 55.950 | 54.809 | **50.944** | **4.798** | |
| **596** | 42.234 | 47.838 | | 47.960 | 52.799 | 48.742 | 51.071 | 51.071 | 52.164 | 54.116 | 39.930 | 40.559 | 45.793 | 45.644 | 39.221 | 40.888 | 45.335 | 51.523 | 49.895 | **47.044** | **4.824** | |
| **604** | 34.296 | 43.985 | | 43.534 | 45.430 | 40.018 | 43.167 | 43.167 | 43.822 | 46.308 | 34.847 | 32.398 | 37.800 | 38.940 | 36.396 | 42.404 | 40.398 | 45.310 | 39.922 | **40.675** | **4.159** | |
| **612** | 31.474 | 37.649 | | 41.266 | 42.063 | 37.254 | 39.478 | 39.478 | 40.199 | 43.178 | 31.294 | 32.033 | 34.675 | 36.230 | 32.316 | 37.087 | 34.610 | 39.514 | 37.990 | **37.099** | **3.706** | |
| **620** | 27.997 | 32.970 | | 33.387 | 34.991 | 32.333 | 34.714 | 34.714 | 32.687 | 35.911 | 26.820 | 27.684 | 30.940 | 27.452 | 28.052 | 30.140 | 31.568 | 32.214 | 34.462 | **31.613** | **2.954** | |
| **628** | 26.244 | 31.858 | | 32.875 | 34.617 | 30.879 | 33.221 | 33.221 | 32.986 | 32.793 | 25.306 | 25.335 | 28.382 | 29.774 | 25.020 | 31.322 | 32.546 | 33.200 | 35.566 | **30.841** | **3.373** | |
| **637** | 21.548 | 23.394 | | 25.269 | 26.505 | 23.062 | 25.385 | 25.385 | 25.614 | 26.898 | 20.455 | 20.730 | 24.483 | 19.906 | 20.276 | 24.098 | 24.473 | 27.178 | 26.889 | **23.975** | **2.459** | |
| **645** | 18.325 | 20.844 | | 21.299 | 22.994 | 20.918 | 21.662 | 21.662 | 22.044 | 21.441 | 17.425 | 16.948 | 19.697 | 19.098 | 17.786 | 18.602 | 19.969 | 22.302 | 22.884 | **20.328** | **1.913** | |
| **653** | 17.066 | 20.697 | | 20.678 | 22.469 | 19.758 | 20.684 | 20.684 | 21.551 | 22.731 | 16.677 | 16.954 | 20.344 | 17.124 | 18.129 | 19.231 | 18.757 | 22.438 | 21.595 | **19.865** | **2.015** | |
| **661** | 16.717 | 18.973 | | 17.597 | 19.969 | 18.674 | 19.734 | 19.734 | 21.554 | 20.918 | 16.292 | 14.893 | 18.345 | 16.607 | 16.751 | 17.612 | 19.670 | 18.345 | 22.154 | **18.586** | **1.949** | |
| **669** | 14.998 | 16.020 | | 16.702 | 17.763 | 15.914 | 17.163 | 17.163 | 17.566 | 17.791 | 14.050 | 13.441 | 15.163 | 15.315 | 14.610 | 17.049 | 17.126 | 17.338 | 16.450 | **16.201** | **1.330** | |
| **678** | 12.630 | 13.741 | | 14.882 | 15.130 | 14.142 | 14.720 | 14.720 | 15.467 | 14.382 | 12.006 | 13.237 | 14.762 | 13.142 | 13.678 | 13.659 | 14.703 | 14.899 | 17.174 | **14.282** | **1.173** | |
| **686** | 12.514 | 13.524 | | 14.406 | 14.321 | 13.842 | 14.485 | 14.485 | 13.821 | 15.893 | 11.362 | 11.757 | 12.028 | 12.226 | 12.856 | 14.559 | 12.924 | 13.794 | 13.812 | **13.478** | **1.183** | |
| **694** | 12.007 | 14.188 | | 13.529 | 14.797 | 13.014 | 14.517 | 14.517 | 14.581 | 14.879 | 12.566 | 11.820 | 13.475 | 11.894 | 10.366 | 13.186 | 14.334 | 12.812 | 14.506 | **13.388** | **1.281** | |
| **702** | 9.546 | 10.414 | | 11.822 | 11.428 | 11.290 | 11.244 | 11.244 | 11.157 | 11.534 | 9.815 | 9.985 | 11.402 | 10.059 | 10.001 | 9.651 | 10.976 | 12.878 | 11.701 | **10.897** | **0.906** | |
| **710** | 8.689 | 9.478 | | 9.654 | 10.656 | 10.220 | 10.306 | 10.306 | 10.465 | 10.636 | 8.955 | 8.846 | 10.429 | 9.619 | 9.249 | 9.114 | 10.158 | 10.222 | 9.487 | **9.805** | **0.649** | |
| **719** | 7.925 | 8.433 | | 7.767 | 8.302 | 8.298 | 8.690 | 8.690 | 8.473 | 8.168 | 7.314 | 8.252 | 8.102 | 7.286 | 7.501 | 8.318 | 7.741 | 7.539 | 8.072 | **8.048** | **0.439** | |
| **727** | 7.222 | 7.506 | | 7.255 | 7.790 | 7.349 | 7.191 | 7.191 | 7.170 | 7.806 | 6.994 | 7.554 | 7.154 | 6.914 | 6.713 | 6.767 | 7.680 | 7.158 | 8.228 | **7.313** | **0.389** | |
| **735** | 6.572 | 6.526 | | 6.750 | 6.484 | 6.710 | 6.588 | 6.588 | 6.605 | 6.515 | 6.606 | 6.101 | 6.498 | 6.541 | 6.596 | 6.393 | 7.077 | 6.613 | 6.797 | **6.587** | **0.193** | |

| **Root extracts** | | | | | | | | | | | | | | | | | | | |
| --- | --- | --- | --- | --- | --- | --- | --- | --- | --- | --- | --- | --- | --- | --- | --- | --- | --- | --- | --- |
|  | **Intensity of emitted fluorescence** | | | | | | | | | | | | | | | | | | |
| **Wavelength nm** | **1** | **2** | **3** | **4** | **5** | **6** | **7** | **8** | **9** | **10** | **11** | **12** | **13** | **14** | **15** | **16** | **17** | **Average** | **SE** |
| **415** | 13.453 | 13.424 | 13.450 | 13.449 | 13.438 | 13.439 | 13.431 | 13.398 | 13.466 | 13.434 | 13.424 | 13.434 | 13.410 | 13.434 | 13.400 | 13.442 | 13.476 | **13.436** | **0.02** |
| **423** | 15.214 | 15.256 | 15.417 | 15.485 | 15.470 | 15.288 | 14.958 | 15.029 | 15.372 | 15.336 | 15.375 | 15.120 | 14.975 | 15.386 | 15.211 | 15.370 | 15.528 | **15.282** | **0.18** |
| **432** | 23.762 | 23.945 | 24.750 | 25.236 | 25.070 | 24.829 | 22.496 | 23.594 | 24.485 | 25.438 | 24.539 | 23.148 | 22.102 | 24.476 | 23.997 | 24.662 | 24.714 | **24.191** | **0.93** |
| **440** | 36.416 | 37.648 | 39.474 | 41.121 | 40.349 | 39.086 | 34.533 | 35.919 | 38.821 | 39.859 | 38.056 | 35.579 | 33.285 | 39.470 | 37.449 | 38.695 | 39.035 | **37.929** | **2.15** |
| **448** | 93.952 | 99.576 | 106.136 | 111.120 | 109.656 | 104.944 | 87.912 | 95.272 | 104.240 | 108.832 | 100.504 | 91.272 | 84.544 | 103.872 | 97.024 | 101.800 | 104.920 | **100.328** | **7.69** |
| **456** | 163.408 | 172.192 | 181.368 | 186.984 | 185.352 | 179.576 | 154.928 | 167.792 | 178.120 | 184.864 | 175.288 | 159.000 | 149.512 | 178.472 | 171.584 | 178.680 | 180.680 | **173.400** | **11.03** |
| **465** | 177.248 | 184.584 | 192.568 | 196.472 | 195.216 | 191.528 | 170.280 | 182.840 | 190.424 | 194.264 | 189.680 | 172.488 | 166.736 | 190.552 | 186.648 | 191.208 | 192.168 | **186.171** | **9.17** |
| **473** | 178.184 | 185.704 | 193.576 | 196.952 | 195.832 | 193.528 | 172.040 | 185.400 | 192.080 | 195.000 | 189.656 | 174.376 | 169.704 | 193.184 | 187.920 | 191.640 | 193.648 | **187.554** | **8.75** |
| **481** | 165.448 | 175.264 | 185.144 | 189.848 | 188.280 | 186.304 | 161.664 | 176.664 | 182.504 | 186.608 | 179.664 | 162.096 | 158.640 | 184.064 | 180.096 | 184.168 | 185.056 | **178.324** | **10.16** |
| **490** | 147.296 | 157.288 | 168.640 | 175.880 | 174.480 | 170.712 | 144.672 | 160.520 | 166.632 | 169.688 | 161.424 | 145.224 | 142.760 | 169.208 | 164.224 | 168.568 | 170.312 | **162.208** | **10.93** |
| **498** | 110.320 | 118.448 | 128.448 | 134.776 | 133.128 | 131.216 | 108.296 | 121.160 | 127.464 | 129.904 | 123.312 | 108.832 | 108.344 | 128.584 | 124.816 | 129.064 | 129.864 | **123.293** | **9.15** |
| **506** | 72.655 | 77.314 | 82.832 | 87.768 | 86.648 | 84.648 | 71.435 | 79.533 | 82.568 | 84.256 | 79.754 | 70.479 | 71.365 | 84.904 | 81.584 | 83.128 | 83.512 | **80.258** | **5.63** |
| **514** | 58.578 | 62.758 | 67.013 | 70.594 | 69.742 | 68.870 | 58.424 | 64.286 | 67.180 | 67.220 | 65.376 | 58.280 | 58.570 | 68.450 | 66.225 | 68.258 | 68.850 | **65.216** | **4.31** |
| **523** | 59.429 | 63.394 | 67.836 | 71.706 | 70.258 | 70.425 | 58.554 | 65.418 | 68.361 | 68.625 | 65.606 | 58.668 | 59.500 | 70.518 | 67.538 | 68.523 | 68.675 | **66.061** | **4.50** |
| **531** | 58.364 | 61.465 | 66.085 | 69.706 | 67.717 | 69.238 | 57.623 | 64.741 | 65.858 | 67.168 | 63.461 | 57.048 | 58.025 | 66.815 | 66.598 | 67.460 | 66.910 | **64.370** | **4.24** |
| **539** | 51.045 | 54.178 | 57.982 | 60.555 | 60.130 | 60.186 | 50.859 | 57.202 | 58.060 | 59.024 | 56.077 | 50.516 | 50.817 | 59.278 | 57.831 | 59.031 | 59.500 | **56.604** | **3.66** |
| **548** | 43.577 | 45.226 | 48.230 | 51.202 | 50.182 | 50.698 | 42.720 | 47.982 | 47.581 | 48.812 | 47.062 | 42.966 | 43.554 | 49.668 | 48.919 | 49.045 | 49.817 | **47.485** | **2.83** |
| **556** | 36.412 | 38.382 | 40.571 | 42.566 | 42.222 | 42.386 | 36.250 | 40.072 | 40.966 | 41.685 | 40.124 | 36.486 | 37.108 | 41.710 | 40.586 | 41.442 | 41.384 | **40.021** | **2.22** |
| **564** | 31.757 | 33.467 | 35.162 | 36.837 | 36.448 | 36.558 | 31.806 | 34.870 | 35.014 | 36.346 | 34.415 | 31.196 | 32.258 | 35.678 | 35.466 | 36.658 | 36.370 | **34.724** | **1.92** |
| **572** | 28.510 | 29.494 | 31.198 | 33.019 | 32.338 | 32.509 | 28.226 | 30.960 | 31.178 | 31.268 | 30.275 | 27.872 | 28.503 | 31.821 | 31.876 | 31.782 | 31.488 | **30.725** | **1.62** |
| **581** | 24.470 | 25.355 | 26.981 | 27.950 | 27.397 | 27.650 | 24.497 | 26.522 | 26.802 | 27.134 | 26.299 | 24.488 | 24.422 | 27.227 | 27.005 | 27.074 | 27.211 | **26.381** | **1.23** |
| **589** | 21.655 | 22.594 | 23.672 | 24.499 | 24.334 | 24.184 | 21.786 | 23.264 | 23.714 | 23.986 | 23.382 | 21.610 | 21.906 | 24.063 | 23.742 | 23.555 | 24.053 | **23.294** | **0.99** |
| **597** | 20.112 | 20.836 | 21.742 | 22.119 | 22.048 | 22.054 | 20.128 | 21.238 | 21.472 | 21.527 | 21.343 | 19.750 | 20.481 | 21.769 | 21.530 | 21.742 | 22.087 | **21.293** | **0.76** |
| **605** | 18.396 | 19.142 | 19.691 | 20.147 | 20.157 | 19.882 | 18.532 | 19.330 | 19.578 | 19.846 | 19.417 | 18.378 | 18.419 | 19.919 | 19.526 | 20.262 | 19.816 | **19.438** | **0.65** |
| **614** | 17.206 | 17.438 | 18.130 | 18.491 | 18.526 | 18.355 | 17.246 | 18.092 | 18.014 | 18.343 | 18.168 | 17.144 | 17.058 | 18.840 | 18.243 | 18.536 | 18.246 | **18.005** | **0.56** |
| **622** | 16.427 | 16.798 | 17.050 | 17.421 | 17.595 | 17.547 | 16.476 | 16.880 | 17.305 | 17.096 | 16.903 | 16.256 | 16.421 | 17.406 | 17.551 | 17.347 | 17.240 | **17.042** | **0.44** |
| **630** | 15.676 | 15.791 | 16.245 | 16.452 | 16.446 | 16.454 | 15.582 | 15.970 | 16.282 | 16.423 | 16.082 | 15.550 | 15.755 | 16.374 | 16.337 | 16.227 | 16.585 | **16.131** | **0.34** |
| **639** | 14.766 | 14.962 | 15.169 | 15.259 | 15.368 | 15.342 | 14.784 | 15.202 | 15.218 | 15.106 | 14.965 | 14.743 | 14.815 | 15.259 | 15.200 | 15.369 | 15.281 | **15.106** | **0.22** |
| **647** | 14.456 | 14.637 | 14.842 | 14.997 | 14.921 | 14.934 | 14.566 | 14.841 | 14.719 | 15.087 | 14.763 | 14.454 | 14.626 | 14.872 | 14.874 | 14.834 | 14.822 | **14.779** | **0.18** |
| **655** | 14.314 | 14.402 | 14.603 | 14.612 | 14.596 | 14.649 | 14.366 | 14.364 | 14.552 | 14.564 | 14.520 | 14.273 | 14.230 | 14.664 | 14.526 | 14.665 | 14.618 | **14.501** | **0.14** |
| **663** | 13.995 | 14.067 | 14.233 | 14.329 | 14.322 | 14.286 | 14.028 | 14.175 | 14.264 | 14.265 | 14.085 | 14.004 | 13.972 | 14.278 | 14.153 | 14.340 | 14.226 | **14.178** | **0.13** |
| **672** | 13.893 | 13.903 | 13.967 | 14.141 | 14.077 | 14.005 | 13.785 | 13.930 | 14.031 | 14.002 | 13.937 | 13.854 | 13.780 | 14.150 | 13.972 | 14.049 | 13.970 | **13.967** | **0.11** |
| **680** | 13.686 | 13.743 | 13.734 | 13.908 | 13.826 | 13.832 | 13.701 | 13.745 | 13.833 | 13.701 | 13.707 | 13.663 | 13.689 | 13.833 | 13.820 | 13.775 | 13.775 | **13.763** | **0.07** |
| **688** | 13.557 | 13.620 | 13.659 | 13.670 | 13.688 | 13.630 | 13.594 | 13.675 | 13.601 | 13.673 | 13.678 | 13.578 | 13.534 | 13.741 | 13.646 | 13.712 | 13.649 | **13.641** | **0.06** |
| **697** | 13.494 | 13.498 | 13.567 | 13.548 | 13.541 | 13.562 | 13.437 | 13.510 | 13.513 | 13.544 | 13.582 | 13.433 | 13.444 | 13.518 | 13.554 | 13.617 | 13.562 | **13.525** | **0.05** |
| **705** | 13.403 | 13.355 | 13.451 | 13.390 | 13.407 | 13.427 | 13.380 | 13.466 | 13.415 | 13.422 | 13.434 | 13.379 | 13.373 | 13.408 | 13.420 | 13.439 | 13.480 | **13.415** | **0.03** |
| **713** | 13.288 | 13.294 | 13.260 | 13.329 | 13.350 | 13.305 | 13.274 | 13.309 | 13.294 | 13.315 | 13.259 | 13.302 | 13.336 | 13.316 | 13.310 | 13.303 | 13.330 | **13.304** | **0.03** |
| **721** | 13.306 | 13.279 | 13.303 | 13.262 | 13.286 | 13.265 | 13.268 | 13.244 | 13.276 | 13.290 | 13.322 | 13.250 | 13.246 | 13.255 | 13.265 | 13.274 | 13.326 | **13.278** | **0.03** |
| **730** | 13.247 | 13.229 | 13.229 | 13.242 | 13.206 | 13.238 | 13.235 | 13.230 | 13.235 | 13.206 | 13.206 | 13.196 | 13.220 | 13.227 | 13.230 | 13.231 | 13.211 | **13.225** | **0.01** |
| **738** | 13.210 | 13.233 | 13.196 | 13.221 | 13.224 | 13.218 | 13.219 | 13.190 | 13.216 | 13.215 | 13.206 | 13.218 | 13.191 | 13.185 | 13.222 | 13.197 | 13.241 | **13.212** | **0.02** |

| **Anthocyanidin-cyanidin chloride** | | | | | | | | | | | | | |  |
| --- | --- | --- | --- | --- | --- | --- | --- | --- | --- | --- | --- | --- | --- | --- |
|  | **Intensity of emitted fluorescence** | | | | | | | | | | | | | |
| **Wavelength nm** | **1** | **2** | **3** | **4** | **5** | **6** | **7** | **8** | **9** | **10** | **11** | **Average** | **SE** | |
| **413** | 9.006 | 8.942 | 8.658 | 8.071 | 8.517 | 9.153 | 8.067 | 7.813 | 8.537 | 8.964 | 9.322 | **8.641** | **0.15** | |
| **421** | 5.204 | 4.842 | 4.330 | 4.185 | 4.318 | 4.711 | 3.897 | 5.055 | 4.231 | 4.728 | 4.411 | **4.537** | **0.40** | |
| **429** | 4.112 | 4.270 | 4.288 | 4.253 | 4.542 | 4.613 | 4.520 | 3.830 | 4.620 | 4.524 | 4.970 | **4.413** | **0.30** | |
| **437** | 4.419 | 4.537 | 4.502 | 4.866 | 3.853 | 4.810 | 4.380 | 4.926 | 4.391 | 4.400 | 4.624 | **4.519** | **0.30** | |
| **445** | 4.743 | 4.991 | 4.968 | 5.035 | 4.638 | 4.564 | 5.391 | 6.047 | 4.892 | 5.550 | 4.528 | **5.032** | **0.47** | |
| **453** | 4.389 | 4.304 | 3.744 | 4.035 | 3.795 | 3.813 | 4.564 | 4.146 | 4.013 | 4.160 | 4.343 | **4.119** | **0.27** | |
| **461** | 3.721 | 4.201 | 3.896 | 3.588 | 4.519 | 4.217 | 3.631 | 4.209 | 3.776 | 3.394 | 4.538 | **3.972** | **0.39** | |
| **469** | 4.178 | 4.021 | 3.968 | 4.032 | 3.729 | 3.937 | 3.665 | 4.033 | 4.194 | 3.592 | 3.726 | **3.916** | **0.21** | |
| **477** | 3.786 | 3.851 | 4.186 | 3.804 | 3.829 | 4.040 | 3.578 | 4.354 | 4.024 | 3.578 | 4.350 | **3.944** | **0.27** | |
| **485** | 3.727 | 3.746 | 3.764 | 4.051 | 4.100 | 3.617 | 4.078 | 4.030 | 3.922 | 4.264 | 3.482 | **3.889** | **0.24** | |
| **493** | 3.550 | 3.680 | 3.643 | 3.745 | 3.635 | 3.368 | 3.522 | 3.799 | 3.668 | 3.612 | 3.868 | **3.645** | **0.14** | |
| **501** | 3.494 | 3.719 | 3.709 | 3.418 | 4.185 | 3.650 | 3.894 | 3.887 | 3.542 | 4.092 | 3.799 | **3.763** | **0.24** | |
| **509** | 3.573 | 3.740 | 3.822 | 4.341 | 3.388 | 3.537 | 3.701 | 3.731 | 3.440 | 3.430 | 3.589 | **3.663** | **0.27** | |
| **517** | 3.637 | 3.746 | 3.694 | 3.915 | 3.344 | 3.704 | 3.338 | 3.544 | 3.650 | 3.924 | 3.528 | **3.639** | **0.19** | |
| **525** | 3.742 | 3.779 | 3.726 | 3.657 | 3.836 | 3.512 | 3.760 | 3.239 | 3.615 | 3.786 | 3.520 | **3.652** | **0.17** | |
| **533** | 3.333 | 3.682 | 3.668 | 3.562 | 3.418 | 3.994 | 3.606 | 3.747 | 3.836 | 3.520 | 3.992 | **3.669** | **0.21** | |
| **541** | 3.550 | 3.722 | 3.629 | 3.573 | 3.554 | 4.099 | 3.905 | 3.555 | 3.461 | 3.890 | 3.629 | **3.688** | **0.20** | |
| **549** | 3.890 | 4.238 | 3.561 | 4.398 | 3.364 | 4.006 | 3.916 | 3.885 | 3.870 | 3.768 | 3.665 | **3.869** | **0.29** | |
| **557** | 4.151 | 4.171 | 4.066 | 4.415 | 4.425 | 3.780 | 4.162 | 4.835 | 3.889 | 3.742 | 3.964 | **4.145** | **0.32** | |
| **565** | 4.474 | 4.803 | 4.433 | 4.099 | 4.308 | 5.204 | 5.168 | 4.286 | 4.941 | 4.314 | 4.812 | **4.622** | **0.38** | |
| **573** | 5.232 | 5.613 | 6.106 | 5.275 | 4.811 | 5.941 | 4.623 | 5.835 | 6.830 | 6.042 | 5.937 | **5.659** | **0.63** | |
| **581** | 5.948 | 6.430 | 7.128 | 7.417 | 6.154 | 6.295 | 6.964 | 7.060 | 7.285 | 7.172 | 6.310 | **6.742** | **0.52** | |
| **589** | 8.765 | 9.532 | 9.333 | 7.707 | 7.715 | 7.694 | 8.651 | 9.291 | 10.348 | 9.156 | 8.645 | **8.803** | **0.85** | |
| **597** | 11.557 | 11.543 | 12.943 | 11.535 | 10.587 | 11.489 | 11.623 | 12.582 | 14.432 | 13.142 | 11.023 | **12.042** | **1.11** | |
| **605** | 14.687 | 15.280 | 15.850 | 15.446 | 12.864 | 14.558 | 17.561 | 15.159 | 15.712 | 16.158 | 14.081 | **15.214** | **1.21** | |
| **613** | 17.869 | 18.479 | 20.376 | 19.559 | 14.462 | 17.919 | 18.296 | 21.615 | 21.541 | 22.246 | 18.632 | **19.181** | **2.23** | |
| **621** | 20.338 | 21.612 | 24.118 | 22.403 | 17.227 | 20.377 | 21.380 | 21.129 | 20.914 | 20.916 | 23.777 | **21.290** | **1.85** | |
| **629** | 27.855 | 28.294 | 30.516 | 26.225 | 24.949 | 28.791 | 29.112 | 30.255 | 27.906 | 31.330 | 27.373 | **28.419** | **1.88** | |
| **637** | 29.989 | 30.262 | 32.136 | 31.157 | 25.065 | 27.397 | 31.271 | 31.398 | 28.749 | 31.544 | 30.173 | **29.922** | **2.11** | |
| **645** | 33.825 | 34.123 | 36.471 | 30.699 | 28.703 | 33.294 | 35.877 | 35.181 | 36.649 | 32.850 | 32.452 | **33.648** | **2.45** | |
| **653** | 36.439 | 38.214 | 38.469 | 36.969 | 34.402 | 34.748 | 34.332 | 38.596 | 38.167 | 41.436 | 36.538 | **37.119** | **2.16** | |
| **661** | 38.940 | 40.311 | 41.766 | 37.832 | 34.198 | 37.473 | 42.539 | 40.459 | 41.649 | 39.514 | 36.635 | **39.210** | **2.51** | |
| **669** | 33.435 | 33.686 | 34.428 | 32.117 | 28.914 | 29.435 | 29.852 | 34.555 | 34.936 | 34.964 | 31.434 | **32.523** | **2.30** | |
| **677** | 31.919 | 33.002 | 30.825 | 31.494 | 26.928 | 27.643 | 31.575 | 33.223 | 36.197 | 30.690 | 30.322 | **31.256** | **2.55** | |
| **685** | 27.788 | 29.562 | 28.145 | 28.012 | 24.682 | 26.237 | 30.704 | 24.651 | 31.310 | 30.382 | 28.673 | **28.195** | **2.28** | |
| **693** | 29.098 | 28.564 | 29.303 | 29.139 | 25.813 | 29.774 | 29.883 | 29.865 | 26.666 | 28.188 | 28.706 | **28.636** | **1.32** | |
| **701** | 22.666 | 23.370 | 24.485 | 23.147 | 21.166 | 22.990 | 28.642 | 23.137 | 21.183 | 23.362 | 20.673 | **23.166** | **2.15** | |
| **709** | 20.500 | 19.004 | 19.329 | 18.787 | 17.671 | 17.403 | 21.723 | 19.868 | 19.273 | 21.200 | 18.518 | **19.389** | **1.36** | |
| **717** | 18.570 | 18.816 | 19.952 | 18.441 | 15.493 | 17.868 | 16.050 | 19.698 | 20.763 | 21.380 | 18.147 | **18.653** | **1.80** | |
| **725** | 16.973 | 18.001 | 16.490 | 17.810 | 16.178 | 15.226 | 18.545 | 15.022 | 17.324 | 16.982 | 14.348 | **16.627** | **1.33** | |
| **733** | 15.165 | 15.163 | 14.910 | 14.648 | 13.014 | 14.629 | 15.564 | 12.324 | 14.938 | 15.126 | 13.581 | **14.460** | **1.03** | |

| **Rutin** | | | | | | | | | | | | | | | | |
| --- | --- | --- | --- | --- | --- | --- | --- | --- | --- | --- | --- | --- | --- | --- | --- | --- |
|  | **Intensity of emitted fluorescence** | | | | | | | | | | | | | | |  |
| **Wavelength nm** | **1** | **2** | **3** | **4** | **5** | **6** | **7** | **8** | **9** | **10** | **11** | **12** | **13** | **Average** | **SE** | |
| **413** | 5.661 | 5.745 | 5.480 | 5.400 | 6.088 | 6.164 | 4.935 | 6.605 | 7.318 | 7.344 | 5.739 | 7.360 | 5.652 | **6.115** | **0.27** | |
| **421** | 6.159 | 6.429 | 6.965 | 5.771 | 7.651 | 7.370 | 6.032 | 7.413 | 8.418 | 8.074 | 6.595 | 7.925 | 6.851 | **7.050** | **0.84** | |
| **429** | 11.746 | 12.117 | 12.934 | 10.849 | 12.916 | 12.478 | 13.555 | 13.176 | 13.838 | 13.684 | 12.907 | 14.066 | 14.028 | **12.946** | **0.95** | |
| **437** | 16.238 | 17.524 | 17.087 | 15.080 | 18.406 | 17.096 | 18.664 | 17.204 | 18.055 | 18.520 | 17.482 | 19.158 | 18.118 | **17.587** | **1.10** | |
| **445** | 21.935 | 22.280 | 20.887 | 20.697 | 23.648 | 23.011 | 23.981 | 22.666 | 24.418 | 21.958 | 23.175 | 24.960 | 24.206 | **22.909** | **1.33** | |
| **453** | 26.276 | 27.394 | 26.715 | 24.654 | 28.727 | 26.749 | 30.144 | 26.216 | 28.172 | 28.261 | 28.778 | 27.287 | 31.747 | **27.778** | **1.84** | |
| **461** | 27.399 | 27.823 | 26.742 | 25.588 | 28.562 | 27.034 | 29.755 | 28.008 | 29.945 | 26.045 | 27.899 | 25.926 | 28.591 | **27.640** | **1.38** | |
| **469** | 25.306 | 25.224 | 23.636 | 23.901 | 25.529 | 25.837 | 27.189 | 24.937 | 24.965 | 23.979 | 26.283 | 23.092 | 26.076 | **25.073** | **1.17** | |
| **477** | 24.503 | 24.985 | 22.219 | 23.527 | 24.651 | 23.585 | 27.083 | 23.274 | 23.857 | 22.802 | 26.209 | 22.009 | 25.543 | **24.173** | **1.51** | |
| **485** | 22.459 | 22.744 | 20.610 | 23.045 | 21.780 | 21.408 | 24.595 | 20.894 | 21.420 | 20.941 | 22.462 | 18.901 | 24.156 | **21.955** | **1.53** | |
| **493** | 17.264 | 17.818 | 15.501 | 17.903 | 17.223 | 17.740 | 19.124 | 16.226 | 16.116 | 15.155 | 19.511 | 14.826 | 17.863 | **17.098** | **1.45** | |
| **501** | 21.211 | 21.443 | 17.747 | 21.139 | 19.010 | 18.981 | 23.293 | 19.170 | 18.251 | 17.773 | 21.027 | 16.814 | 22.062 | **19.840** | **1.97** | |
| **509** | 23.910 | 24.672 | 21.284 | 25.228 | 23.111 | 22.064 | 26.083 | 23.266 | 21.894 | 20.175 | 26.670 | 18.719 | 25.403 | **23.268** | **2.38** | |
| **517** | 22.803 | 24.323 | 20.368 | 23.037 | 23.434 | 21.198 | 25.539 | 23.381 | 20.941 | 20.626 | 25.609 | 19.569 | 24.473 | **22.716** | **2.01** | |
| **525** | 27.159 | 27.701 | 24.529 | 27.778 | 26.341 | 25.126 | 28.472 | 25.143 | 24.738 | 23.460 | 28.391 | 22.451 | 27.435 | **26.056** | **1.95** | |
| **533** | 29.579 | 30.904 | 29.381 | 29.745 | 29.575 | 28.992 | 33.468 | 27.969 | 28.843 | 27.042 | 31.968 | 23.024 | 30.577 | **29.313** | **2.51** | |
| **541** | 32.041 | 34.656 | 31.152 | 32.285 | 32.779 | 32.352 | 35.112 | 31.094 | 32.208 | 28.990 | 34.253 | 27.538 | 33.024 | **32.114** | **2.12** | |
| **549** | 41.884 | 44.276 | 40.806 | 39.439 | 42.873 | 40.601 | 45.804 | 40.268 | 40.707 | 38.217 | 42.836 | 35.345 | 41.350 | **41.108** | **2.65** | |
| **557** | 47.355 | 50.369 | 47.667 | 46.748 | 48.871 | 47.346 | 50.075 | 45.990 | 46.567 | 43.597 | 53.046 | 40.794 | 44.908 | **47.180** | **3.12** | |
| **565** | 68.435 | 69.270 | 67.796 | 64.551 | 68.677 | 68.070 | 68.394 | 65.024 | 66.450 | 60.124 | 74.663 | 57.964 | 65.008 | **66.494** | **4.21** | |
| **573** | 78.321 | 82.657 | 78.248 | 70.855 | 80.576 | 78.683 | 80.041 | 76.833 | 78.420 | 71.738 | 85.079 | 67.080 | 75.522 | **77.235** | **4.94** | |
| **581** | 91.241 | 93.907 | 89.595 | 84.012 | 93.853 | 92.559 | 91.157 | 89.244 | 87.910 | 82.655 | 95.692 | 77.343 | 84.817 | **88.768** | **5.28** | |
| **589** | 110.520 | 112.890 | 110.713 | 103.559 | 110.537 | 113.502 | 112.417 | 107.897 | 107.123 | 101.649 | 113.236 | 96.489 | 101.603 | **107.857** | **5.44** | |
| **597** | 115.290 | 120.969 | 115.319 | 100.333 | 116.685 | 116.080 | 115.547 | 111.822 | 112.570 | 105.882 | 122.546 | 100.798 | 107.133 | **112.383** | **6.99** | |
| **605** | 134.662 | 140.950 | 135.249 | 119.950 | 137.046 | 137.818 | 133.167 | 132.894 | 130.227 | 125.727 | 142.938 | 121.205 | 127.157 | **132.230** | **7.11** | |
| **613** | 127.469 | 130.957 | 129.052 | 112.810 | 131.167 | 131.004 | 129.639 | 125.497 | 126.928 | 121.337 | 134.909 | 114.015 | 119.039 | **125.679** | **6.88** | |
| **621** | 128.913 | 136.783 | 135.629 | 115.383 | 135.637 | 139.596 | 129.587 | 131.342 | 131.780 | 125.097 | 136.909 | 119.398 | 125.053 | **130.085** | **7.25** | |
| **629** | 129.116 | 138.357 | 136.401 | 114.846 | 138.624 | 140.002 | 131.247 | 130.860 | 133.639 | 126.140 | 140.955 | 125.235 | 126.421 | **131.680** | **7.44** | |
| **637** | 125.385 | 129.256 | 125.821 | 108.488 | 127.902 | 132.635 | 129.385 | 127.808 | 126.568 | 120.423 | 135.680 | 118.813 | 118.864 | **125.156** | **7.06** | |
| **645** | 119.193 | 125.571 | 125.375 | 103.321 | 123.873 | 127.412 | 123.398 | 120.116 | 123.244 | 117.453 | 129.350 | 113.251 | 115.593 | **120.550** | **6.99** | |
| **653** | 108.133 | 113.906 | 111.688 | 92.087 | 114.976 | 119.859 | 114.615 | 112.658 | 111.475 | 108.763 | 115.818 | 105.171 | 105.641 | **110.368** | **6.90** | |
| **661** | 94.376 | 101.974 | 98.829 | 83.839 | 100.200 | 105.871 | 97.810 | 99.800 | 101.680 | 94.933 | 102.246 | 95.642 | 93.706 | **97.762** | **5.51** | |
| **669** | 80.305 | 85.867 | 85.857 | 69.177 | 84.050 | 91.644 | 85.542 | 84.464 | 88.893 | 84.033 | 89.083 | 81.379 | 81.637 | **83.995** | **5.50** | |
| **677** | 79.031 | 84.961 | 86.895 | 67.948 | 85.267 | 89.688 | 85.624 | 84.305 | 86.934 | 82.523 | 88.185 | 80.405 | 80.306 | **83.236** | **5.59** | |
| **685** | 65.376 | 68.402 | 69.329 | 54.112 | 68.136 | 71.182 | 70.384 | 71.332 | 68.990 | 70.154 | 71.610 | 68.291 | 66.868 | **68.013** | **4.55** | |
| **693** | 53.021 | 58.547 | 57.774 | 42.708 | 57.291 | 61.413 | 57.572 | 57.427 | 58.978 | 56.946 | 59.242 | 57.110 | 56.351 | **56.491** | **4.56** | |
| **701** | 45.071 | 48.598 | 50.907 | 39.515 | 48.951 | 49.650 | 49.345 | 49.610 | 53.270 | 49.444 | 51.493 | 47.969 | 47.325 | **48.550** | **3.37** | |
| **709** | 36.000 | 39.408 | 40.179 | 30.672 | 40.556 | 42.109 | 40.029 | 40.616 | 41.778 | 40.154 | 41.647 | 41.408 | 38.789 | **39.488** | **3.09** | |
| **717** | 32.918 | 34.576 | 35.276 | 29.200 | 35.411 | 39.463 | 36.955 | 38.043 | 38.226 | 36.323 | 36.190 | 35.501 | 35.736 | **35.678** | **2.58** | |
| **725** | 25.093 | 28.532 | 29.115 | 21.588 | 29.411 | 29.728 | 30.489 | 28.594 | 29.593 | 28.150 | 30.154 | 28.560 | 28.559 | **28.274** | **2.41** | |
| **733** | 20.366 | 21.221 | 22.917 | 15.659 | 23.564 | 24.038 | 24.047 | 22.611 | 25.278 | 23.302 | 23.953 | 22.714 | 23.738 | **22.570** | **2.43** | |

| **Ferulic-hydroxyl cinnamic acid** | | | | | | | | | | | | | | | | | | | |
| --- | --- | --- | --- | --- | --- | --- | --- | --- | --- | --- | --- | --- | --- | --- | --- | --- | --- | --- | --- |
|  |  | | **Intensity of emitted fluorescence** | | | | | | | | | | | | | | | |  |
| **Wavelength nm** | **1** | **2** | | **3** | **4** | **5** | **6** | **7** | **8** | **9** | **10** | **11** | **12** | **13** | **14** | **15** | **Average** | **SE** | |
| **415** | 30.520 | 28.280 | | 37.082 | 33.382 | 32.417 | 36.485 | 28.507 | 25.268 | 29.153 | 22.510 | 30.223 | 28.085 | 29.312 | 30.333 | 34.441 | **30.400** | **1.01** | |
| **423** | 64.250 | 61.402 | | 77.114 | 70.079 | 64.843 | 75.798 | 60.716 | 58.543 | 64.581 | 54.223 | 64.720 | 63.669 | 60.391 | 67.373 | 65.136 | **64.856** | **1.55** | |
| **431** | 98.841 | 89.915 | | 111.826 | 102.351 | 95.422 | 109.181 | 91.123 | 84.470 | 90.660 | 79.837 | 92.256 | 92.666 | 85.256 | 92.045 | 101.678 | **94.502** | **2.30** | |
| **440** | 107.176 | 102.760 | | 113.104 | 110.395 | 107.052 | 116.916 | 97.596 | 93.312 | 102.507 | 88.243 | 111.089 | 98.980 | 101.938 | 98.502 | 111.010 | **104.039** | **2.04** | |
| **448** | 110.300 | 104.578 | | 122.854 | 112.485 | 107.976 | 120.954 | 108.868 | 96.123 | 112.059 | 92.442 | 111.308 | 104.698 | 104.485 | 99.065 | 110.427 | **107.908** | **2.12** | |
| **456** | 112.018 | 105.534 | | 120.725 | 119.750 | 109.116 | 122.883 | 103.782 | 97.097 | 114.892 | 93.359 | 119.002 | 103.421 | 105.940 | 102.219 | 111.234 | **109.398** | **2.29** | |
| **464** | 107.839 | 103.167 | | 121.094 | 112.465 | 107.766 | 124.222 | 104.493 | 96.454 | 103.118 | 89.825 | 113.429 | 102.474 | 100.596 | 100.711 | 109.790 | **106.496** | **2.30** | |
| **472** | 106.960 | 99.936 | | 117.370 | 105.215 | 103.224 | 114.212 | 103.350 | 90.039 | 102.567 | 81.873 | 108.288 | 95.145 | 93.724 | 93.179 | 100.390 | **101.031** | **2.39** | |
| **481** | 96.299 | 92.529 | | 106.963 | 104.169 | 100.692 | 105.056 | 97.318 | 82.449 | 95.552 | 76.761 | 105.318 | 92.428 | 91.169 | 92.726 | 98.597 | **95.868** | **2.18** | |
| **489** | 90.448 | 82.359 | | 99.246 | 96.092 | 87.800 | 100.747 | 85.074 | 80.283 | 83.084 | 75.008 | 99.253 | 84.269 | 86.735 | 80.726 | 92.719 | **88.256** | **2.04** | |
| **497** | 82.771 | 76.320 | | 90.526 | 81.099 | 78.030 | 90.054 | 79.450 | 70.226 | 74.015 | 65.092 | 85.677 | 73.501 | 73.276 | 71.438 | 80.542 | **78.134** | **1.87** | |
| **505** | 67.811 | 67.604 | | 77.132 | 77.360 | 70.271 | 74.668 | 67.991 | 59.176 | 62.246 | 57.896 | 76.402 | 64.053 | 62.774 | 67.831 | 67.851 | **68.071** | **1.61** | |
| **513** | 60.967 | 58.722 | | 69.268 | 63.162 | 64.692 | 72.347 | 59.857 | 52.885 | 59.355 | 52.430 | 63.596 | 57.297 | 55.528 | 61.990 | 68.322 | **61.361** | **1.49** | |
| **522** | 53.774 | 50.572 | | 60.804 | 54.061 | 51.163 | 61.901 | 48.857 | 46.407 | 50.606 | 44.363 | 55.119 | 49.215 | 46.934 | 46.090 | 52.129 | **51.466** | **1.31** | |
| **530** | 43.800 | 44.993 | | 46.988 | 48.868 | 45.267 | 50.291 | 43.129 | 40.168 | 41.813 | 35.231 | 46.710 | 39.915 | 43.902 | 39.294 | 40.519 | **43.392** | **1.03** | |
| **538** | 38.429 | 37.086 | | 40.720 | 41.050 | 34.957 | 41.082 | 35.931 | 29.354 | 40.212 | 28.968 | 37.568 | 34.874 | 36.068 | 28.791 | 40.075 | **36.344** | **1.12** | |
| **546** | 29.616 | 30.860 | | 40.074 | 33.342 | 28.890 | 36.258 | 28.550 | 27.761 | 29.222 | 22.382 | 33.489 | 29.294 | 27.254 | 31.015 | 31.119 | **30.608** | **1.06** | |
| **554** | 25.826 | 26.642 | | 31.491 | 28.853 | 24.839 | 29.651 | 25.146 | 24.024 | 27.768 | 20.865 | 29.256 | 23.867 | 25.105 | 25.990 | 26.251 | **26.372** | **0.70** | |
| **563** | 21.747 | 21.069 | | 23.392 | 25.311 | 19.529 | 24.064 | 18.693 | 17.997 | 17.956 | 16.980 | 21.630 | 18.424 | 20.868 | 19.229 | 20.115 | **20.467** | **0.63** | |
| **571** | 17.593 | 18.212 | | 21.174 | 21.864 | 19.617 | 20.901 | 18.711 | 17.908 | 20.887 | 18.434 | 21.196 | 17.538 | 17.714 | 15.637 | 19.905 | **19.153** | **0.46** | |
| **579** | 14.691 | 15.387 | | 16.814 | 15.840 | 16.677 | 18.454 | 13.140 | 13.769 | 12.158 | 12.386 | 18.514 | 14.464 | 14.028 | 15.980 | 13.231 | **15.036** | **0.52** | |
| **587** | 12.984 | 12.599 | | 14.985 | 15.000 | 13.230 | 14.003 | 10.908 | 10.698 | 12.330 | 10.275 | 12.655 | 11.653 | 12.261 | 12.214 | 12.417 | **12.547** | **0.36** | |
| **596** | 9.857 | 10.835 | | 11.303 | 12.075 | 9.105 | 9.763 | 10.235 | 8.299 | 12.374 | 8.008 | 10.675 | 10.883 | 10.427 | 8.945 | 10.658 | **10.229** | **0.33** | |
| **604** | 9.889 | 8.686 | | 9.392 | 10.257 | 7.804 | 11.278 | 8.716 | 8.207 | 9.729 | 6.394 | 10.097 | 8.656 | 10.538 | 8.338 | 9.695 | **9.179** | **0.32** | |
| **612** | 7.543 | 7.683 | | 7.226 | 8.103 | 8.174 | 7.112 | 7.112 | 6.496 | 8.212 | 7.677 | 7.536 | 6.498 | 7.692 | 7.149 | 7.136 | **7.423** | **0.14** | |
| **620** | 5.804 | 6.542 | | 6.382 | 6.954 | 7.245 | 6.390 | 6.278 | 5.415 | 7.192 | 5.143 | 5.933 | 6.110 | 5.925 | 6.915 | 7.153 | **6.359** | **0.17** | |
| **628** | 5.210 | 5.695 | | 5.129 | 5.366 | 6.028 | 6.620 | 3.808 | 5.449 | 5.197 | 5.187 | 5.149 | 5.541 | 5.011 | 5.502 | 4.132 | **5.268** | **0.17** | |
| **637** | 4.679 | 5.041 | | 5.650 | 5.250 | 4.142 | 5.194 | 4.315 | 3.911 | 5.222 | 3.896 | 5.444 | 4.220 | 5.263 | 4.065 | 4.366 | **4.711** | **0.16** | |
| **645** | 3.957 | 4.352 | | 4.457 | 3.956 | 4.402 | 3.898 | 5.567 | 4.252 | 3.424 | 3.139 | 5.300 | 3.724 | 3.517 | 3.612 | 4.434 | **4.133** | **0.17** | |
| **653** | 3.777 | 3.586 | | 3.923 | 4.156 | 3.378 | 3.763 | 3.307 | 3.097 | 3.571 | 3.044 | 3.385 | 3.680 | 3.985 | 3.731 | 3.508 | **3.593** | **0.08** | |
| **661** | 3.474 | 3.537 | | 3.459 | 3.860 | 3.329 | 3.378 | 3.206 | 3.856 | 3.547 | 3.323 | 3.695 | 3.329 | 3.709 | 3.881 | 2.831 | **3.494** | **0.07** | |
| **669** | 3.271 | 3.354 | | 3.404 | 3.300 | 3.454 | 2.758 | 3.327 | 3.018 | 2.773 | 2.287 | 3.226 | 2.720 | 2.910 | 2.886 | 2.936 | **3.042** | **0.08** | |
| **678** | 3.013 | 2.757 | | 2.464 | 3.088 | 3.159 | 2.480 | 2.777 | 2.604 | 2.399 | 2.558 | 2.583 | 3.106 | 2.889 | 3.055 | 2.858 | **2.786** | **0.07** | |
| **686** | 2.765 | 2.568 | | 2.357 | 2.364 | 2.404 | 2.929 | 2.734 | 2.567 | 2.586 | 2.410 | 2.424 | 2.480 | 2.718 | 3.323 | 2.342 | **2.598** | **0.07** | |
| **694** | 2.674 | 2.550 | | 2.519 | 2.274 | 2.978 | 2.349 | 2.897 | 2.365 | 2.783 | 2.466 | 2.419 | 2.565 | 2.312 | 2.801 | 2.173 | **2.542** | **0.06** | |
| **702** | 2.304 | 2.499 | | 2.538 | 2.605 | 2.320 | 2.278 | 2.375 | 2.646 | 2.793 | 2.191 | 2.342 | 2.330 | 2.365 | 2.886 | 2.878 | **2.490** | **0.06** | |
| **710** | 2.220 | 2.222 | | 2.196 | 3.099 | 2.460 | 2.651 | 2.393 | 2.058 | 2.064 | 2.243 | 2.516 | 2.241 | 2.158 | 2.219 | 2.190 | **2.328** | **0.07** | |
| **719** | 2.133 | 2.428 | | 2.511 | 2.452 | 2.471 | 2.117 | 2.464 | 2.315 | 2.734 | 2.454 | 2.655 | 2.312 | 2.254 | 2.557 | 2.102 | **2.397** | **0.05** | |
| **727** | 2.355 | 2.264 | | 2.050 | 2.206 | 2.241 | 2.033 | 2.499 | 2.215 | 2.020 | 2.092 | 2.395 | 2.348 | 2.278 | 2.000 | 2.247 | **2.216** | **0.04** | |
| **735** | 2.169 | 2.121 | | 2.139 | 2.110 | 2.049 | 2.270 | 2.069 | 2.013 | 2.207 | 2.363 | 2.218 | 2.081 | 2.147 | 2.144 | 2.014 | **2.141** | **0.02** | |

| **4-methyl umbelliferone coumarin** | | | | | | | | | | | | | | | | | |  |
| --- | --- | --- | --- | --- | --- | --- | --- | --- | --- | --- | --- | --- | --- | --- | --- | --- | --- | --- |
|  | **Intensity of emitted fluorescence** | | | | | | | | | | | | | | | | | |
| **Wavelength nm** | **1** | **2** | **3** | **4** | **5** | **6** | **7** | **8** | **9** | **10** | **11** | **12** | **13** | **14** | **15** | **Average** | **SE** | |
| **415** | 13.453 | 13.424 | 13.450 | 13.449 | 13.438 | 13.439 | 13.431 | 13.398 | 13.466 | 13.434 | 13.424 | 13.434 | 13.410 | 13.434 | 13.400 | **13.436** | **0.02** | |
| **423** | 15.214 | 15.256 | 15.417 | 15.485 | 15.470 | 15.288 | 14.958 | 15.029 | 15.372 | 15.336 | 15.375 | 15.120 | 14.975 | 15.386 | 15.211 | **15.282** | **0.18** | |
| **432** | 23.762 | 23.945 | 24.750 | 25.236 | 25.070 | 24.829 | 22.496 | 23.594 | 24.485 | 25.438 | 24.539 | 23.148 | 22.102 | 24.476 | 23.997 | **24.191** | **0.93** | |
| **440** | 36.416 | 37.648 | 39.474 | 41.121 | 40.349 | 39.086 | 34.533 | 35.919 | 38.821 | 39.859 | 38.056 | 35.579 | 33.285 | 39.470 | 37.449 | **37.929** | **2.15** | |
| **448** | 93.952 | 99.576 | 106.136 | 111.120 | 109.656 | 104.944 | 87.912 | 95.272 | 104.240 | 108.832 | 100.504 | 91.272 | 84.544 | 103.872 | 97.024 | **100.328** | **7.69** | |
| **456** | 163.408 | 172.192 | 181.368 | 186.984 | 185.352 | 179.576 | 154.928 | 167.792 | 178.120 | 184.864 | 175.288 | 159.000 | 149.512 | 178.472 | 171.584 | **173.400** | **11.03** | |
| **465** | 177.248 | 184.584 | 192.568 | 196.472 | 195.216 | 191.528 | 170.280 | 182.840 | 190.424 | 194.264 | 189.680 | 172.488 | 166.736 | 190.552 | 186.648 | **186.171** | **9.17** | |
| **473** | 178.184 | 185.704 | 193.576 | 196.952 | 195.832 | 193.528 | 172.040 | 185.400 | 192.080 | 195.000 | 189.656 | 174.376 | 169.704 | 193.184 | 187.920 | **187.554** | **8.75** | |
| **481** | 165.448 | 175.264 | 185.144 | 189.848 | 188.280 | 186.304 | 161.664 | 176.664 | 182.504 | 186.608 | 179.664 | 162.096 | 158.640 | 184.064 | 180.096 | **178.324** | **10.16** | |
| **490** | 147.296 | 157.288 | 168.640 | 175.880 | 174.480 | 170.712 | 144.672 | 160.520 | 166.632 | 169.688 | 161.424 | 145.224 | 142.760 | 169.208 | 164.224 | **162.208** | **10.93** | |
| **498** | 110.320 | 118.448 | 128.448 | 134.776 | 133.128 | 131.216 | 108.296 | 121.160 | 127.464 | 129.904 | 123.312 | 108.832 | 108.344 | 128.584 | 124.816 | **123.293** | **9.15** | |
| **506** | 72.655 | 77.314 | 82.832 | 87.768 | 86.648 | 84.648 | 71.435 | 79.533 | 82.568 | 84.256 | 79.754 | 70.479 | 71.365 | 84.904 | 81.584 | **80.258** | **5.63** | |
| **514** | 58.578 | 62.758 | 67.013 | 70.594 | 69.742 | 68.870 | 58.424 | 64.286 | 67.180 | 67.220 | 65.376 | 58.280 | 58.570 | 68.450 | 66.225 | **65.216** | **4.31** | |
| **523** | 59.429 | 63.394 | 67.836 | 71.706 | 70.258 | 70.425 | 58.554 | 65.418 | 68.361 | 68.625 | 65.606 | 58.668 | 59.500 | 70.518 | 67.538 | **66.061** | **4.50** | |
| **531** | 58.364 | 61.465 | 66.085 | 69.706 | 67.717 | 69.238 | 57.623 | 64.741 | 65.858 | 67.168 | 63.461 | 57.048 | 58.025 | 66.815 | 66.598 | **64.370** | **4.24** | |
| **539** | 51.045 | 54.178 | 57.982 | 60.555 | 60.130 | 60.186 | 50.859 | 57.202 | 58.060 | 59.024 | 56.077 | 50.516 | 50.817 | 59.278 | 57.831 | **56.604** | **3.66** | |
| **548** | 43.577 | 45.226 | 48.230 | 51.202 | 50.182 | 50.698 | 42.720 | 47.982 | 47.581 | 48.812 | 47.062 | 42.966 | 43.554 | 49.668 | 48.919 | **47.485** | **2.83** | |
| **556** | 36.412 | 38.382 | 40.571 | 42.566 | 42.222 | 42.386 | 36.250 | 40.072 | 40.966 | 41.685 | 40.124 | 36.486 | 37.108 | 41.710 | 40.586 | **40.021** | **2.22** | |
| **564** | 31.757 | 33.467 | 35.162 | 36.837 | 36.448 | 36.558 | 31.806 | 34.870 | 35.014 | 36.346 | 34.415 | 31.196 | 32.258 | 35.678 | 35.466 | **34.724** | **1.92** | |
| **572** | 28.510 | 29.494 | 31.198 | 33.019 | 32.338 | 32.509 | 28.226 | 30.960 | 31.178 | 31.268 | 30.275 | 27.872 | 28.503 | 31.821 | 31.876 | **30.725** | **1.62** | |
| **581** | 24.470 | 25.355 | 26.981 | 27.950 | 27.397 | 27.650 | 24.497 | 26.522 | 26.802 | 27.134 | 26.299 | 24.488 | 24.422 | 27.227 | 27.005 | **26.381** | **1.23** | |
| **589** | 21.655 | 22.594 | 23.672 | 24.499 | 24.334 | 24.184 | 21.786 | 23.264 | 23.714 | 23.986 | 23.382 | 21.610 | 21.906 | 24.063 | 23.742 | **23.294** | **0.99** | |
| **597** | 20.112 | 20.836 | 21.742 | 22.119 | 22.048 | 22.054 | 20.128 | 21.238 | 21.472 | 21.527 | 21.343 | 19.750 | 20.481 | 21.769 | 21.530 | **21.293** | **0.76** | |
| **605** | 18.396 | 19.142 | 19.691 | 20.147 | 20.157 | 19.882 | 18.532 | 19.330 | 19.578 | 19.846 | 19.417 | 18.378 | 18.419 | 19.919 | 19.526 | **19.438** | **0.65** | |
| **614** | 17.206 | 17.438 | 18.130 | 18.491 | 18.526 | 18.355 | 17.246 | 18.092 | 18.014 | 18.343 | 18.168 | 17.144 | 17.058 | 18.840 | 18.243 | **18.005** | **0.56** | |
| **622** | 16.427 | 16.798 | 17.050 | 17.421 | 17.595 | 17.547 | 16.476 | 16.880 | 17.305 | 17.096 | 16.903 | 16.256 | 16.421 | 17.406 | 17.551 | **17.042** | **0.44** | |
| **630** | 15.676 | 15.791 | 16.245 | 16.452 | 16.446 | 16.454 | 15.582 | 15.970 | 16.282 | 16.423 | 16.082 | 15.550 | 15.755 | 16.374 | 16.337 | **16.131** | **0.34** | |
| **639** | 14.766 | 14.962 | 15.169 | 15.259 | 15.368 | 15.342 | 14.784 | 15.202 | 15.218 | 15.106 | 14.965 | 14.743 | 14.815 | 15.259 | 15.200 | **15.106** | **0.22** | |
| **647** | 14.456 | 14.637 | 14.842 | 14.997 | 14.921 | 14.934 | 14.566 | 14.841 | 14.719 | 15.087 | 14.763 | 14.454 | 14.626 | 14.872 | 14.874 | **14.779** | **0.18** | |
| **655** | 14.314 | 14.402 | 14.603 | 14.612 | 14.596 | 14.649 | 14.366 | 14.364 | 14.552 | 14.564 | 14.520 | 14.273 | 14.230 | 14.664 | 14.526 | **14.501** | **0.14** | |
| **663** | 13.995 | 14.067 | 14.233 | 14.329 | 14.322 | 14.286 | 14.028 | 14.175 | 14.264 | 14.265 | 14.085 | 14.004 | 13.972 | 14.278 | 14.153 | **14.178** | **0.13** | |
| **672** | 13.893 | 13.903 | 13.967 | 14.141 | 14.077 | 14.005 | 13.785 | 13.930 | 14.031 | 14.002 | 13.937 | 13.854 | 13.780 | 14.150 | 13.972 | **13.967** | **0.11** | |
| **680** | 13.686 | 13.743 | 13.734 | 13.908 | 13.826 | 13.832 | 13.701 | 13.745 | 13.833 | 13.701 | 13.707 | 13.663 | 13.689 | 13.833 | 13.820 | **13.763** | **0.07** | |
| **688** | 13.557 | 13.620 | 13.659 | 13.670 | 13.688 | 13.630 | 13.594 | 13.675 | 13.601 | 13.673 | 13.678 | 13.578 | 13.534 | 13.741 | 13.646 | **13.641** | **0.06** | |
| **697** | 13.494 | 13.498 | 13.567 | 13.548 | 13.541 | 13.562 | 13.437 | 13.510 | 13.513 | 13.544 | 13.582 | 13.433 | 13.444 | 13.518 | 13.554 | **13.525** | **0.05** | |
| **705** | 13.403 | 13.355 | 13.451 | 13.390 | 13.407 | 13.427 | 13.380 | 13.466 | 13.415 | 13.422 | 13.434 | 13.379 | 13.373 | 13.408 | 13.420 | **13.415** | **0.03** | |
| **713** | 13.288 | 13.294 | 13.260 | 13.329 | 13.350 | 13.305 | 13.274 | 13.309 | 13.294 | 13.315 | 13.259 | 13.302 | 13.336 | 13.316 | 13.310 | **13.304** | **0.03** | |
| **721** | 13.306 | 13.279 | 13.303 | 13.262 | 13.286 | 13.265 | 13.268 | 13.244 | 13.276 | 13.290 | 13.322 | 13.250 | 13.246 | 13.255 | 13.265 | **13.278** | **0.03** | |
| **730** | 13.247 | 13.229 | 13.229 | 13.242 | 13.206 | 13.238 | 13.235 | 13.230 | 13.235 | 13.206 | 13.206 | 13.196 | 13.220 | 13.227 | 13.230 | **13.225** | **0.01** | |
| **738** | 13.210 | 13.233 | 13.196 | 13.221 | 13.224 | 13.218 | 13.219 | 13.190 | 13.216 | 13.215 | 13.206 | 13.218 | 13.191 | 13.185 | 13.222 | **13.212** | **0.02** | |
